# Association Between Circulating CD4^+^ T Cell Methylation Signatures of Network-Oriented SOCS3 Gene and Hemodynamics in Patients Suffering Pulmonary Arterial Hypertension

**DOI:** 10.1007/s12265-022-10294-1

**Published:** 2022-08-12

**Authors:** Giuditta Benincasa, Bradley A. Maron, Ornella Affinito, Michele D’Alto, Monica Franzese, Paola Argiento, Concetta Schiano, Emanuele Romeo, Paola Bontempo, Paolo Golino, Liberato Berrino, Joseph Loscalzo, Claudio Napoli

**Affiliations:** 1 Department of Advanced Medical and Surgical Sciences (DAMSS), University of Campania “Luigi Vanvitelli”, 80138 Naples, Italy; 2 Division of Cardiovascular Medicine, Department of Medicine, Brigham and Women’s Hospital, MB Boston, USA; 3 Harvard Medical School, Boston, MA USA; 4 IRCCS SDN, Naples, Italy; 5 Department of Cardiology, Monaldi Hospital, University of Campania “Luigi Vanvitelli”, Naples, Italy; 6 Department of Precision Medicine, University of Campania “Luigi Vanvitelli”, Naples, Italy; 7 Department of Experimental Medicine, University of Campania “Luigi Vanvitelli”, Naples, Italy

**Keywords:** Pulmonary Arterial Hypertension, DNA Methylation, CD4^+^ T cells, Network Analysis, Hemodynamic Parameters

## Abstract

Pathogenic DNA methylation changes may be involved in pulmonary arterial hypertension (PAH) onset and its progression, but there is no data on potential associations with patient-derived hemodynamic parameters. The reduced representation bisulfite sequencing (RRBS) platform identified *N* = 631 differentially methylated CpG sites which annotated to *N* = 408 genes (DMGs) in circulating CD4^+^ T cells isolated from PAH patients vs. healthy controls (CTRLs). A promoter-restricted network analysis established the PAH subnetwork that included 5 hub DMGs (*SOCS3*, *GNAS*, *ITGAL*, *NCOR2*, *NFIC*) and 5 non-hub DMGs (*NR4A2*, *GRM2*, *PGK1*, *STMN1*, *LIMS2*). The functional analysis revealed that the *SOCS3* gene was the most recurrent among the top ten significant pathways enriching the PAH subnetwork, including the growth hormone receptor and the interleukin-6 signaling. Correlation analysis showed that the promoter methylation levels of each network-oriented DMG were associated individually with hemodynamic parameters. In particular, *SOCS3* hypomethylation was negatively associated with right atrial pressure (RAP) and positively associated with cardiac index (CI) (*|r|*≥ 0.6). A significant upregulation of the *SOCS3*, *ITGAL*, *NFIC*, *NCOR2*, and *PGK1* mRNA levels (qRT-PCR) in peripheral blood mononuclear cells from PAH patients vs. CTRLs was found (*P* ≤ 0.05). By immunoblotting, a significant upregulation of the SOCS3 protein was confirmed in PAH patients vs*.* CTRLs (*P* < 0.01). This is the first network-oriented study which integrates circulating CD4^+^ T cell DNA methylation signatures, hemodynamic parameters, and validation experiments in PAH patients at first diagnosis or early follow-up. Our data suggests that *SOCS3* gene might be involved in PAH pathogenesis and serve as potential prognostic biomarker.

**Graphical abstract:**

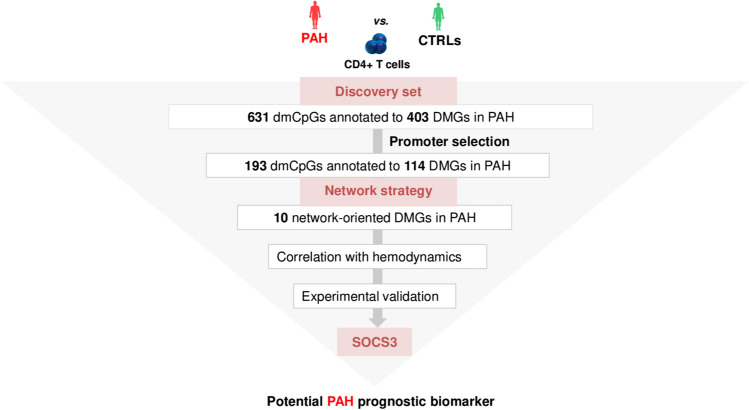

**Supplementary Information:**

The online version contains supplementary material available at 10.1007/s12265-022-10294-1.

## Introduction

Pulmonary arterial hypertension (PAH) is a highly morbid cardiopulmonary disease associated with decreased lifespan despite the availability of vasodilator and other therapies [1–4]. In clinical trials and in practice, treatment response is variable across different PAH subgroups and individual patients [1–4]. This is ascribed to a high heterogeneity in the molecular basis of vascular and cardiac remodeling that underlies PAH, for which no specific biomarkers are established yet [1–4]. The complex nature of PAH suggests that epigenetic-sensitive mechanisms, mainly guided by DNA methylation, are key players underlying a progressive pulmonary vascular remodeling which leads to increased vascular resistance and a concomitant right ventricle remodeling [1]. DNA methylation is a covalent enzymatic modification mediated by DNA methyltransferase which add one or more methyl groups at the 5′-carbon of cytosine bases localized in 5′-CpG-3′ dinucleotides [5]. These latter motifs are most abundant in CpG island (CGI) regions annotated to promoters which can regulate the rate of gene expression at transcriptional level. Generally, there is an inverse relationship between the grade of DNA methylation into the CGI-promoter regions and gene activity, for which DNA hypermethylation is prone to silence gene expression, whereas DNA hypomethylation is transcriptionally more permissive [5]. Previous clinical evidence showed that alterations in DNA methylation signatures were linked to lipid metabolism, incomplete penetrance, and pro-inflammatory pathways [1, 6] which were strongly implicated in the PAH pathogenesis [7, 8]. However, no previous study investigated DNA methylation changes and their potential associations with hemodynamic parameters in PAH patients.

Thus, we leveraged the large CLEOPAHTRA trial (NCT04282434 at clinicaltrials.gov) of well-phenotypes patients to test the hypothesis that PAH patients are distinct from healthy controls by virtue of circulating CD4^+^ T cell DNA methylation signatures, which, in turn, may be relevant for phenotyping and prognostication of affected patients. The goal of our study was to apply a network analysis to patient-derived CD4^+^ T cell methylation profiles to discover novel potential pathogenic mechanisms in patients with PAH at time of diagnosis or early follow-up. Despite the well-known role of monocytes and myeloid cells in PAH response [7], CD4^+^ T cells have been understudied and are now emerging as key players in pulmonary vascular remodeling both in experimental [9–12] and clinical studies [13–16]. Furthermore, based on our previous experience [17, 18], we choose circulating CD4^+^ T cells because of their utility in capturing DNA methylation signatures and molecular pathways mirroring a potential clinical significance.

Here, we perform a novel research program integrating clinical epigenetics and network medicine analytic approaches to advance precision medicine in PAH. Findings from this study have direct translational potential ranging from biomarker discovery and validation to therapeutic target identification that can be studied further in future investigations.

## Material and Methods

### Ethics Statement

This study was approved by the Local Ethics Committee at University of Campania “L. Vanvitelli” Naples, Italy (N. protocol 800/2019). All individuals in the study provided written informed consent for the collection of samples and subsequent analysis. This study was conducted according to the principles and guidelines expressed in the Declaration of Helsinki.

### Study Population

Between April 2019 and January 2020, we enrolled in the CLEOPAHTRA trial (NCT04282434) a total of *N* = 25 consecutive patients with Pulmonary Hypertension (PAH) WHO Groups I who were diagnosed by right heart catheterization (RHC) at the Pulmonary Hypertension Centre of Monaldi Hospital, University of Campania “L. Vanvitelli” (Naples, Italy), according to diagnostic criteria at that time [19]. Specifically, PAH was diagnosed by mean pulmonary arterial pressure (mPAP) ≥ 25 mmHg at rest or mPAP ≥ 30 mmHg with exercise, pulmonary capillary wedge pressure (PCWP) ≤ 15 mmHg, and pulmonary vascular resistance (PVR) ≥ 240 dynes-sec-cm-5 (e.g., ≥ 3.0 wood units). Patients were grouped in idiopathic PAH (IPAH, *N* = 13), PAH associated with congenital heart disease (PAH-CHD, *N* = 5), PAH associated with systemic sclerosis (PAH-SSc, *N* = 5), and PAH associated with portal hypertension (POPH, *N* = 2). PAH patients were stratified using a simplified risk assessment tool that quantifies the number of low-risk criteria (NYHA/WHO: I–II, 6MWD > 440 m, RAP < 8 mmHg, CI > 2.5 L/min/m^2^). Patients with low (3–4 parameters), intermediate (2 parameters), and high (1–0 parameters) risk features have an estimated 1-year risk of death of < 5%, 5–10%, and > 10%, respectively. Patients who meet the criteria for inclusion into PH WHO Groups II, III, IV, or V were excluded from the study. To avoid confounder effects, patients with known history of cancer, active infections, and chronic or immune-mediated diseases were not enrolled. A total of *N* = 16 volunteer blood donors served as healthy controls (CTRLs) and were recruited from the U.O.C. Divisione di Immunologia Clinica, Immunoematologia, Medicina Trasfusionale e Immunologia dei Trapianti, Dipartimento di Medicina Interna e Specialistica, AOU “L. Vanvitelli” University of Campania (Naples, Italy). Clinical characteristics of PAH patients are summarized in Supplementary Table 1.

### Flowchart for Discovery and Validation of DNA Methylation Signatures

As previously described [17, 18], we harvested approximately 10–15 mL of peripheral blood from the jugular vein of *N* = 25 PAH patients during RHC and the antecubital vein of *N* = 16 CTRLs during blood donation. Within 2 h, we collected peripheral blood mononuclear cells (PBMCs), isolated CD4^+^ T cells, and extracted genomic DNA (gDNA). Based on PBMC recovery, we also stored some cellular aliquots for successive validation experiments (Data Supplement).

**Fig. 1:**
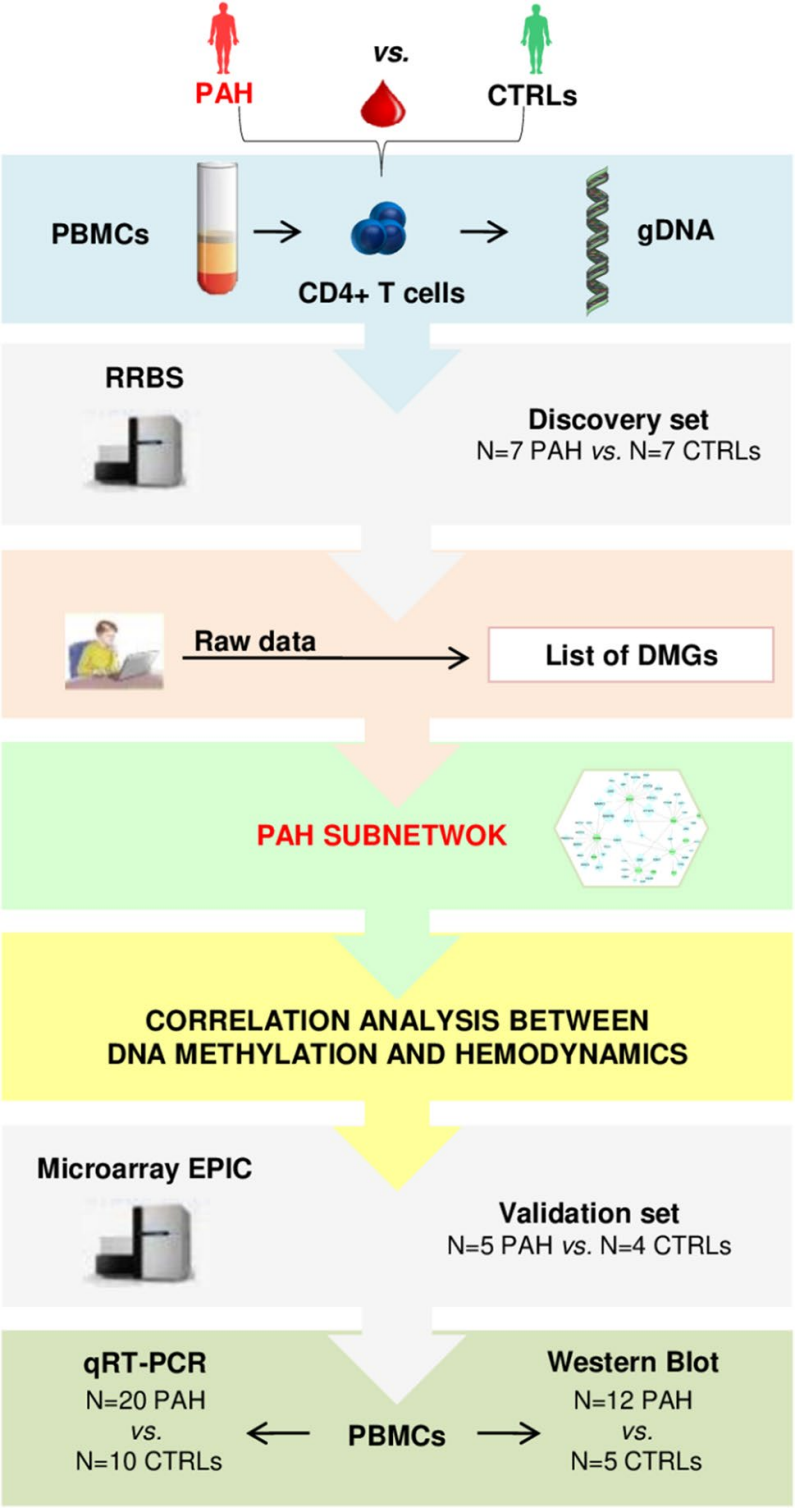
Study design. This flowchart shows the network-oriented strategy used to build the PAH subnetwork via integrating circulating CD4^+^ T cell DNA methylation signatures and hemodynamic parameters. Abbreviations: CTRLs, controls; DMGs, differentially methylated genes; gDNA, genomic DNA; PAH, pulmonary arterial hypertension; PBMCs, peripheral blood mononuclear cells; RRBS, reduced representation bisulfite sequencing

The flowchart for discovery and validation of DNA methylation signatures is shown in the Fig. 1. As a discovery set, *N* = 7 PAH patients vs*. N* = 7 CTRLs were selected for reduced representation bisulfite sequencing (RRBS), which was performed at the Genomix4Life S.r.l. (Salerno, Italy) (https://www.genomix4life.com/it/). To profile genome-wide DNA methylation signatures in the discovery set, we selected the subset of gDNA samples which was of highest quality and highest yield (≥ 2.5–3.0 μg). Then, raw data were analyzed via network analysis to obtain the PAH subnetwork (or PAH interactome) which was successively validated in an independent set of *N* = 5 PAH patients vs*. N* = 4 CTRLs. For validation of network-oriented DNA methylation signatures, we chose the Infinium Human Methylation EPIC BeadChip platform which was performed at the DKFZ Genomics and Proteomics Core Facility (GPCF) in Heidelberg (Germany) (https://dktk.dkfz.de/en/sites/heidelberg/core_facilities) (Data Supplement). Successively, the functional effects of DNA methylation changes were validated in PBMCs using traditional molecular biology techniques, such as qRT-PCR and Western blotting.

### DNA Sequence Processing and Alignment

RRBS raw reads were assessed for quality using FastQC (v011.8, Babraham Bioinformatics, UK) and trimmed using TrimGalore (v0.6.3, Babraham Bioinformatics, UK). Cytosine methylation calls was performed using the Bismark methylation extractor (Supplementary Table 2). Data from Infinium Human Methylation EPIC BeadChip processing were quantile-normalized using the function normalize.quantiles from the Bioconductor package “preprocessCore” (Data Supplement). Raw data have been deposited in the NCBI Gene Expression Omnibus (GEO) database under the accession number GSE165360 (https://www.ncbi.nlm.nih.gov/geo/query/acc.cgi?acc=GSE165360).

### qRT-PCR

TRIzol solution (Thermo Fischer Scientific, USA) was used to extract total RNA from frozen PBMCs of *N* = 20 PAH vs*. N* = 10 CTRLs according to the manufacturer’s instructions. Oligonucleotide sequences are reported in the Supplementary Table 3. The relative mRNA expression levels were measured using the CFX96 touch real-time PCR detection system (BioRad Laboratories, Ltd, USA) (Data Supplement). Expression values were analyzed with the delta Ct (Δct) method using the equation Δct = Ct reference-Ct target. The Δct values of the target genes were normalized to that of the housekeeping gene encoding RPS18 [20]. Each sample was analyzed in duplicate, and relative expression data are given as Fold Change (FC, 2^−ΔΔct^).

### Western Blot

Western blot analysis was performed using frozen PBMCs from *N* = 12 PAH patients vs. *N* = 5 CTRLs. The membranes were incubated with both mouse anti-human anti-SOCS3 (OriGene, #TA502991) at a dilution 1:1000 and mouse anti-human anti-GAPDH at a dilution 1:5000 (Santa-Cruz #sc-365062) overnight at 4 °C and next incubated with peroxidase-labeled secondary antibody and visualized using the ECL detection system (Data Supplement). GAPDH protein served as loading control and was detected on the same membranes for normalizing the signals.

### Bioinformatic Analysis and Data Visualization

#### CpG Differential Methylation Analysis

We identified differentially methylated CpGs (dmCpGs) and annotated genes by comparing the CpG site methylation status in patients with PAH vs. CTRLs using the R package methylKit (v1.10.0) [21], after removing any potential batch effects. Coverage values between samples were normalized by default, and read coverage per base (Supplementary Figs. 1 and 2) was calculated. We then defined as dmCpGs those CpG sites with more than 20% methylation differences (|ΔM|) and *q* value < 0.05, after applying logistic regression using the SLIM method to correct the *p* value for multiple hypothesis testing.

#### Annotation of Differentially Methylated CpG Sites (dmCpGs)

The R package ChIPseeker (v1.20.0) [22] provided detailed annotation about the percentage of dmCpGs in PAH vs*.* CTRLs, which was located in promoters, coding sequences, introns, distal intergenic regions, and 5′ and 3′ untranslated regions (UTRs). Next, annotation of CGIs and CpG shores was obtained from the UCSC website (http://genome.ucsc.edu/; hg38). For clarity, we defined genes annotated to dmCpGs as differentially methylated genes (DMGs).

### Network Analysis

#### Lung-Specific DNA Methylation-PPI Subnetworks

We used the NetworkAnalyst 3.0 tool (http://www.networkanalyst.ca) [23] to build two unique DNA methylation-protein-protein interaction (PPI) subnetworks in PAH. Irrespective from their genomic localization, we mapped separately *N* = 230 hypermethylated and *N* = 178 hypomethylated DMGs (defined as “seed genes”) into the lung-specific PPI network from the DifferentialNet database. In order to identify hub DMGs, we used the *centrality degree* as network topological measure. We defined hubs those DMGs having *centrality degree* ≥ 10.

#### Promoter-Restricted PAH Subnetwork

We built a PPI network between DMGs annotated to promoters (*N* = 114) and known PAH-related genes as per the DisGeNET database (*N* = 172) [24] by using Cytoscape v.3.7.2 [25] and the associated application ReactomeFIViz [26]. Briefly, we first downloaded the list of known PAH-related genes by searching the DisGeNET website using the following keywords: “idiopathic pulmonary arterial hypertension,” “pulmonary arterial hypertension associated with portal hypertension,” and “pulmonary arterial hypertension associated with connective tissue disease.” Each of the PAH-related genes was annotated with supporting publications. We removed genes that were annotated with only one publication to rule out potential false positives. Next, we constructed a PPI network linking DMGs and known PAH-related genes. In order to select hub DMGs with the strongest number of links to their known PAH-related interactors, we chose the *centrality* *degree* ≥ 10 and extracted a subnetwork for each of hub DMGs. Finally, each subnetwork was depicted using Cytoscape’s merge function [27].

### Statistical Analysis

All analyses were performed using the R statistical package (version 3.6.2) (www.cran.r-project.org). The Shapiro–Wilk normality test was used to assess normality of data. The F test was used to evaluate the homogeneity of variance. Statistical significance was computed using two- tailed Student’s *T*-test if variables fulfilled the assumptions of a parametric test; otherwise, the Wilcoxon rank sum exact test was used. Correlation analysis (Pearson’s correlation or Spearman’s correlation) and linear regression were used to evaluate possible associations between hemodynamic parameters and DNA methylation. Statistical tests were considered significant if the *p* value ≤ 0.05. 

### Power Analysis

For the sample size calculation, we considered the total number of annotated genes and the proportion of non-DMGs. We used the R package “ssizeRNA” (version 1.3.1) which gives the power vs. sample size curve estimated by a simulation method using a negative binomial distribution. We determined the sample size with the following parameters: an achieved power of 0.8 at FDR = 0.05. We determined the sample size and assessed the power for detecting DMGs. This analysis revealed that our total sample size of *N *= 14 patients for *N *= 408 DMGs provided an estimated power of 0.74 at FDR = 0.05, given the dispersion of the data, as reported in the Supplementary Fig. 3.

## Results

Clinical characteristics of PAH patients vs*.* CTRLs enrolled in the discovery set are described in Data Supplement and are summarized in Supplementary Table 4. Briefly, we identified 631 dmCpGs (|ΔM|≥ 20%, q-val < 0.05), of which 65% (*N* = 410) were hypermethylated and 35% (*N* = 221) were hypomethylated in PAH patients vs*.* CTRLs (Supplementary Fig. 4A). The majority of the dmCpGs were located within annotated distal intergenic regions (38.54% vs. 31.22% in hypermethylated and hypomethylated clusters, respectively) followed by promoters, defined as ± 1 kb to the transcription start site (TSS) (22.93% vs. 23.53% in hypermethylated and hypomethylated clusters, respectively) (Supplementary Fig. 4B–C). Manhattan plot showed that most dmCpGs were localized on chromosomes 20 (~ 11%) and 7 (~ 7%) (Supplementary Fig. 5A). When interrogating the distribution of the dmCpGs in the context of CGIs and CpG shores, most dmCpGs were found in CGIs associated with promoters (*N* = 116/251, 46%) (Supplementary Fig. 5B). For brevity, the top 40 hypermethylated and hypomethylated dmCpGs are shown in Supplementary Tables 5 and 6, respectively. Our analysis showed *N* = 230 hypermethylated and *N* = 178 hypomethylated DMGs; rarely, DMGs showed both hypermethylation and hypomethylation in their genic regions (5 genes; 0.01%). The DMGs were classified with respect to gene type (Supplementary Fig. 5C–D) and ranked in descending order as: protein-coding genes > long non-coding genes > microRNAs > pseudogenes > unannotated nucleotides.

### Global Network Analysis

The hypermethylated lung-specific DNA methylation-PPI subnetwork included 867 nodes, 996 edges, and 84 seeds (Supplementary Fig. 6). We identified *N* = 28 hub DMGs of which 32% (*N* = 9/28) was annotated to promoter regions (Supplementary Table 7). These included *RPTOR* [28], *CTBP2* [29], and *PGK1* [30] genes, which have been implicated previously in the pathogenesis of PAH. By contrast, *NCOR2*, *NR4A2*, *PKP1*, *DNMT3B*, *SH3BP4*, and *CLEC4G* genes were in the network but have not been reported previously as associated with PAH, thereby suggesting that these are novel epigenetic mediators of PAH. The hypomethylated lung-specific DNA methylation-PPI subnetwork retrieved 763 nodes, 882 edges, and 68 seeds (Supplementary Fig. 7). Among the hub DMGs (*N* = 25), the major percentage (36%, *N* = 9/25) was annotated to promoter regions (Supplementary Table 8)**.** Previous evidence showed that *SOCS3* [31], *BSG* [32], *DLC1* [33], and *GNAS* [34] were potentially involved in PAH, whereas *DPPA4*, *LAMA5*, *SF3A1*, and *RNF166* had no previously recognized association. Globally, these results supported our successive promoter-restricted network analysis.

### PAH Subnetwork

**Fig. 2:**
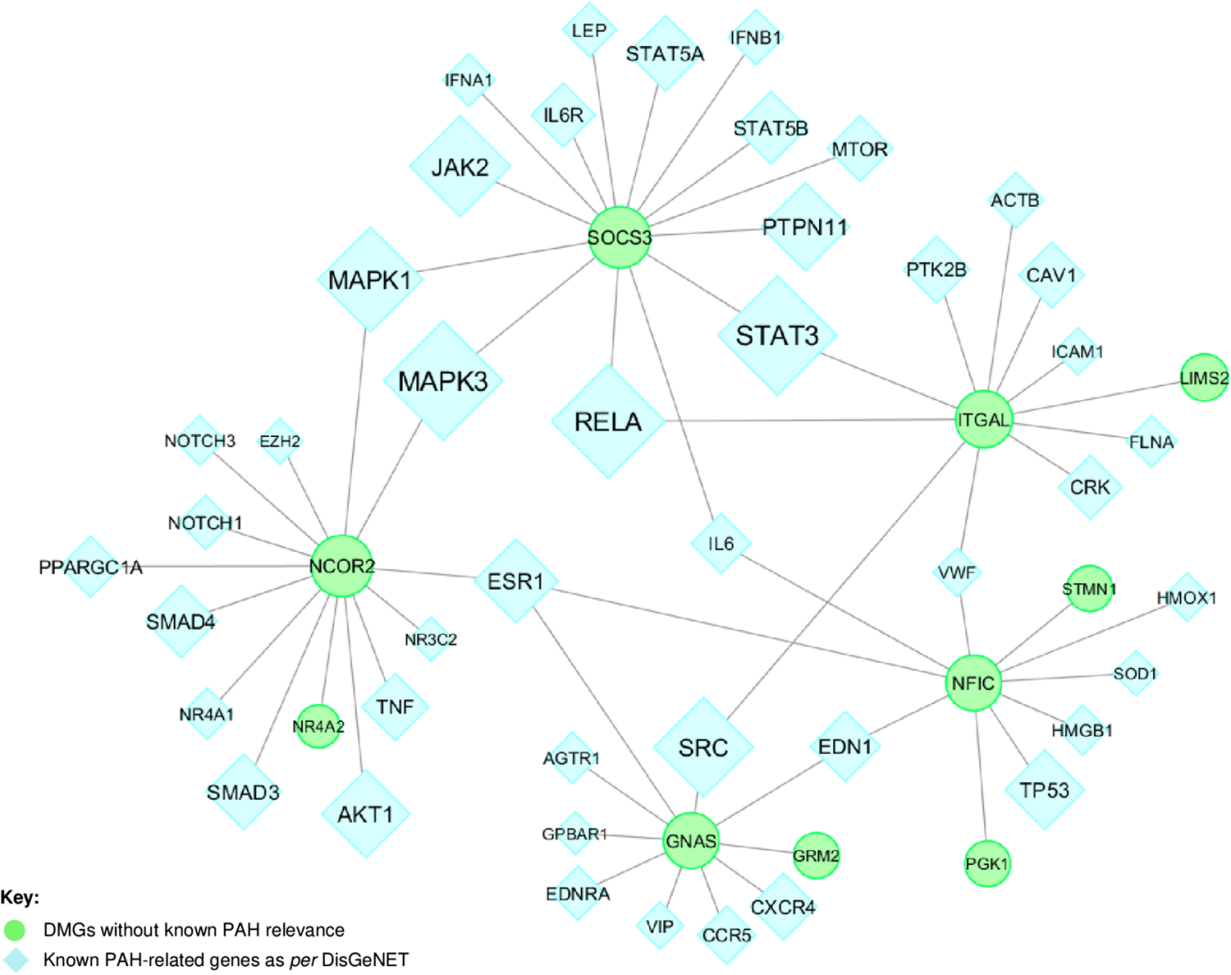
The PAH subnetwork. The interactome shows 5 hub DMGs and 5 non-hub DMGs (green circles) linked through physical interactions (grey links) to specific known PAH-related genes as *per* DisGeNET (light blue diamonds). The size of both circles and diamonds is scaled according to the *centrality* *degree* and *betweenness* *centrality* measures. Original source: Cytoscape v.3.7.2 software

**Fig. 3:**
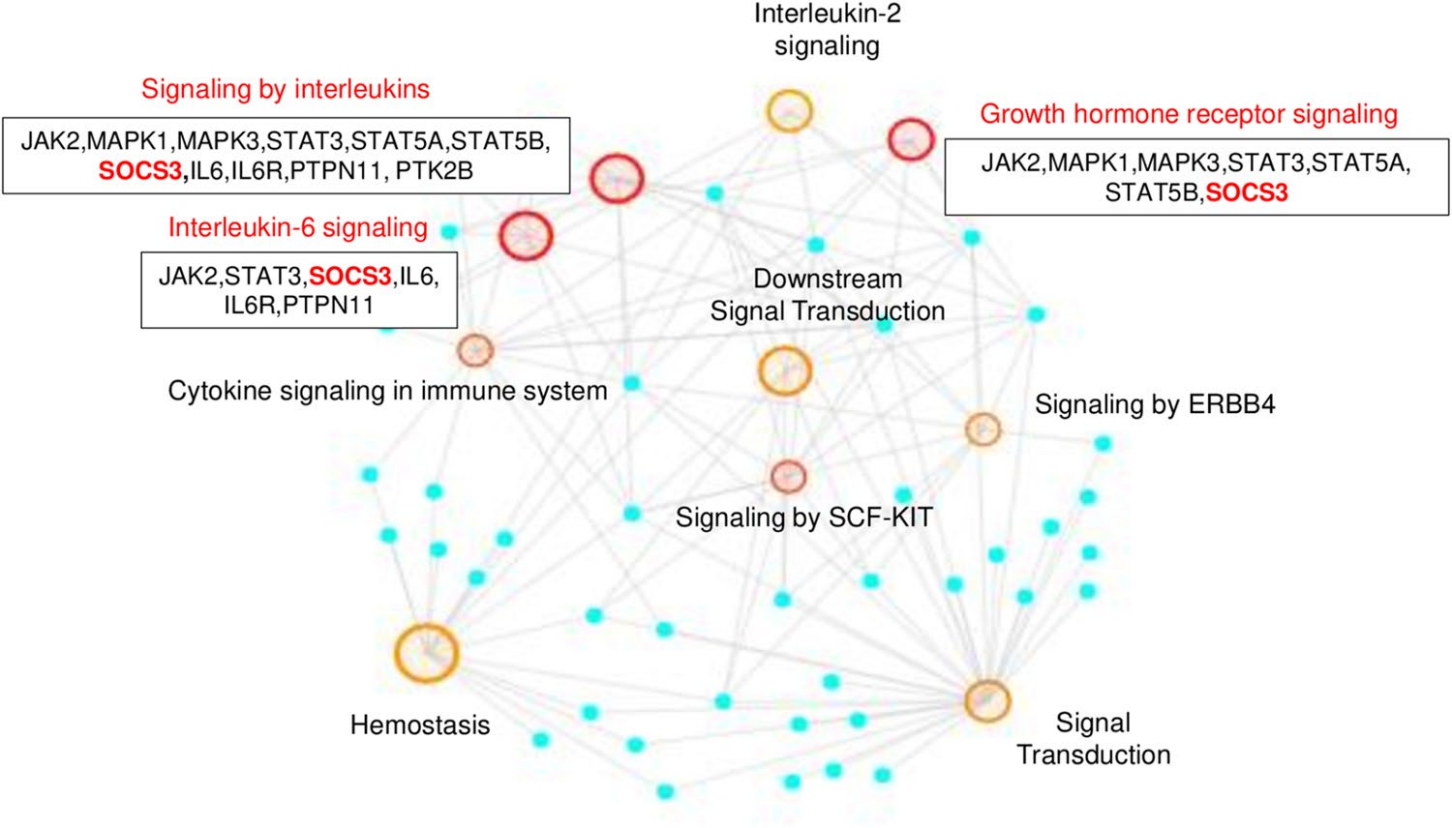
Overrepresentation analysis (ORA). This figure shows the top ten significant pathways enriched by the genes of the PAH subnetwork through REACTOME database. Each pathway is indicated by a red or orange circle according to their *p* value (red circles correspond to the most significant *p* values). The circle size corresponds to the number of genes enriching that pathway. The small light blue circles represent the input gene list (54 nodes) and are located in the network periphery. ORA counts the number of genes shared by an input gene set and each annotated gene set, and applies a statistical test (the cumulative hyper-geometric test) to calculate the statistical significance of the overlap. A *p* value cutoff (*p* < 0.05) is then applied to select the annotated gene sets that have statistically significant overlaps with the input gene set. The IL-6 signaling axis, the growth hormone receptor signaling, and the interleukin signaling (highlighted in red) reached the highest statistical significance. The network-oriented *SOCS3* gene was the most recurrent in the top significant pathways. Original source: NetworkAnalyst 3.0 software

Building “PAH-specific subnetworks” represents a powerful bioinformatic strategy to identify novel potential candidate genes and pathways involved in PAH pathogenesis [35, 36]. The core assumption of this analysis was that a DMG can interact with many known PAH-related genes and, therefore, may also be functionally relevant to the development of PAH. Our PAH subnetwork (Fig. 2) was characterized by 54 nodes and 59 edges in which PPIs linked 5 hub DMGs (*SOCS3*, *ITGAL*, *GNAS*, *NCOR2*, *NFIC*) to known PAH-related genes. In addition, 5 (non-hub) DMGs (*NR4A2*, *GRM2, PGK1, STMN1*, *LIMS2*) were linked to hubs (Supplementary Table 9). We suggest that these 10 network-oriented DMGs may affect or be affected themselves by the activities of their interacting partners with subsequent functional implications in the pathogenesis of PAH. Among the 10 network-oriented DMGs, none had PAH relevance as per DisGeNET; however, *PGK1* [30], *SOCS3* [31], and *GNAS* [34] genes were previously implicated in the pathogenesis of PAH or PAH drug responsiveness. The overrepresentation analysis (ORA) highlighted that the growth hormone receptor-, the interleukin-, and mainly the interleukin-6 (IL-6)- signaling were the most significant pathways of the PAH subnetwork (red circles) (Fig. 3). The inspection of genes enriching the top ten significant pathways (Supplementary Table 10) revealed that the *SOCS3* gene was the most recurrent than other network-oriented DMGs.

### Validation Set of Experiments

To assess the robustness of RRBS, we validated the methylation trends of our 10 network-oriented DMGs through two Infinium Human Methylation EPIC BeadChip arrays with the same content of probes in an independent set of *N* = 5 PAH patients and *N* = 4 CTRLs. Clinical characteristics of PAH patients are summarized in Supplementary Table 11. Starting with overlapping CpGs between the two arrays, we considered only those that confirmed the RRBS trend for each network-oriented DMG (Supplementary Table 12). We observed that hypermethylation of *NCOR2* and *NFIC* genes was confirmed in approximately 54% and 48% of detected CpGs, respectively, whereas analysis of *SOCS3*, *ITGAL*, and *GNAS* genes confirmed hypomethylation of approximately 69%, 79%, and 38% of detected CpGs, respectively. In addition, hypermethylation of *NR4A2*, *GRM2*, and *PGK1* genes was confirmed in approximately 64%, 65%, and 88% of detected CpGs, respectively, while hypomethylation of *STMN1* and *LIMS2* genes was confirmed in approximately 50% and 54% of detected CpGs, respectively. The CpG methylation distributions for the 10 network-oriented DMGs are shown in Supplementary Fig. 8.

### Network-Oriented Promoter DNA Methylation Levels Associate with Patient-Level Hemodynamics

**Fig. 4:**
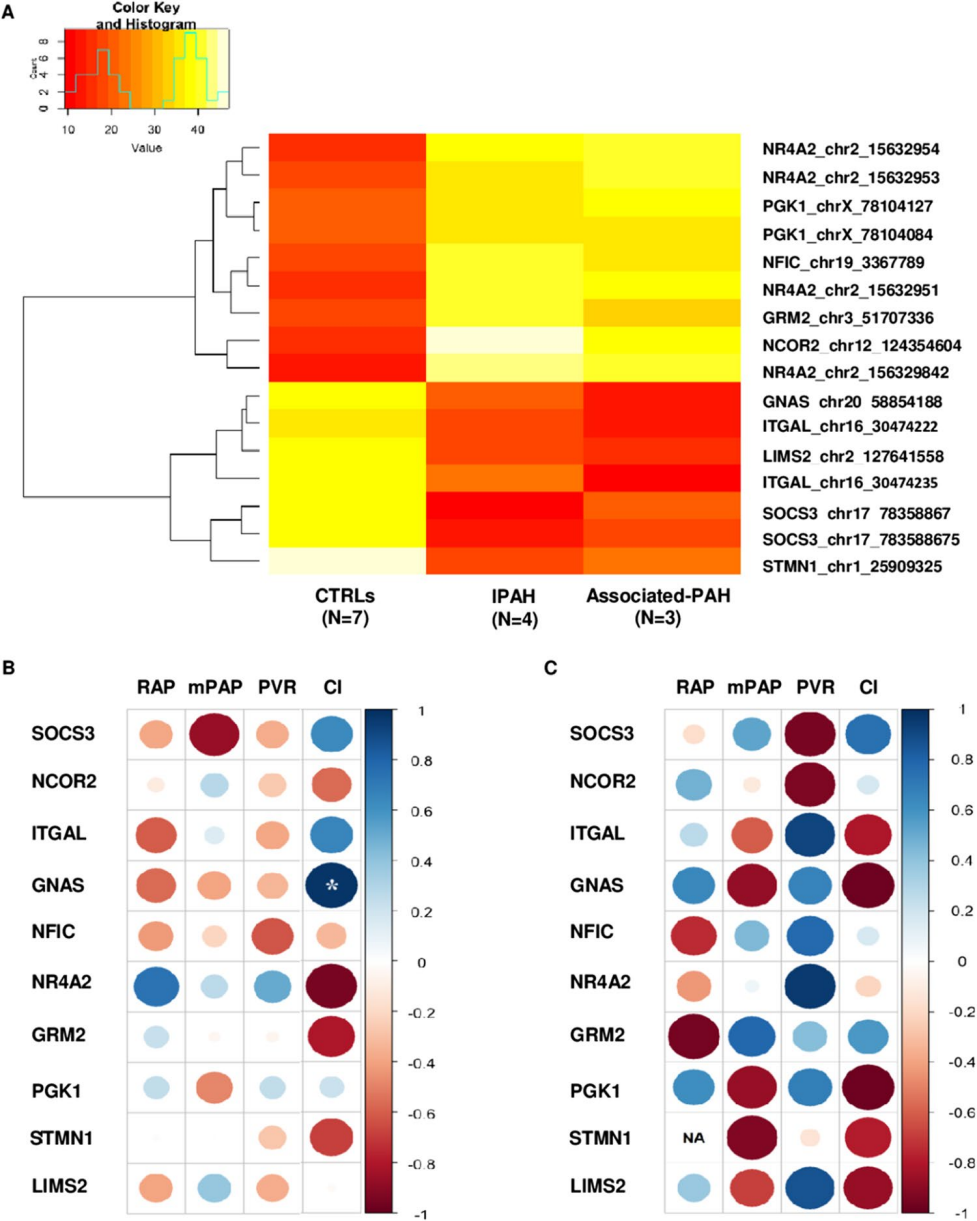
Association between network-oriented promoter DNA methylation levels and hemodynamic parameters. **A** Unsupervised hierarchical clustering shows the mean methylation of the dmCpGs associated with the hub and non-hub DMGs. DNA methylation values of each dmCpG (rows) are averaged within the pre-classified group (column), including CTRLs (*N* = 7) vs*.* IPAH (*N* = 4) and Associated-PAH (*N* = 3). The color key indicates DNA methylation status; red/orange represents hypomethylated DMGs and yellow/white represents hypermethylated DMGs. Correlograms show the Pearson’s correlation between promoter DNA methylation levels of each network-oriented DMG and hemodynamic parameters in IPAH (**B)** or Associated-PAH (**C**). Circle size is scaled by the correlation coefficient. Blue and red colors designate, respectively, positive and negative correlations. The asterisk (*) indicates significant correlations (*p* ≤ 0.05). Abbreviations: PAH, pulmonary arterial hypertension; CI, cardiac index; ITGAL, integrin subunit alpha L; GNAS guanine nucleotide binding protein (G protein), alpha stimulating activity; GRM2, glutamate metabotropic receptor 2; LIMS2, LIM zinc finger domain containing 2; mPAP, mean pulmonary arterial pressure; NCOR2, nuclear receptor corepressor 2; NFIC, nuclear factor I C; NR4A2, nuclear receptor subfamily 4 group A member 2; PGK1, phosphoglycerate kinase 1; PVR, pulmonary vascular pressure; RAP, right atrial pressure; SOCS3, suppressor of cytokine signaling 3; STMN1, stathmin

In Fig. 4A, the heatmap shows the methylation status (hypomethylation in red and hypermethylation in yellow) of significant dmCpGs (*N* = 16) which were annotated to network-oriented promoters discriminating CTRLs vs. PAH patients. However, each of them failed in discriminating IPAH vs. Associated-PAH patients. Correlation analysis was performed to evaluate whether promoter methylation levels were associated individually with hemodynamic parameters, including mPAP, PVR, right atrial pressure (RAP), and cardiac index (CI). We observed that each of these 10 network-oriented DMGs was strongly correlated (*|r|*≥ 0.6) with at least one of the four hemodynamic parameters both in IPAH (Fig. 4B) and Associated-PAH (Fig. 4C), even if a statistical significance was reached only for the *GNAS* gene. Besides, we observed that a panel of DMGs was negatively correlated (red signal) with RAP and positively correlated (blu signal) with CI (or vice versa), which are the most robust indicators of right ventricle function and prognosis. This occurred in 5/10 network-oriented DMGs (*SOCS3*, *ITGAL*, *GNAS*, *NR4A2*, and *GRM2*) in IPAH (Fig. 4B) and 7/10 network-oriented DMGs in Associated-PAH (*SOCS3*, *ITGAL*, *GNAS*, *NFIC*, *GRM2*, *PGK1*, and *LIMS2*) (Fig. 4C). We also noted that *SOCS3* hypomethylation was negatively associated with RAP (red signal) and positively associated with CI (blu signal) in both IPAH (|r|= -0.4 and |r|= 0.6, respectively) and Associated-PAH (|r|= − 0.2 and |r|= 0.7, respectively) patients suggesting a potential prognostic biomarker.

### Transcriptional Profiles of Network-Oriented *SOCS3*, *ITGAL*, *NFIC*, *NCOR2*, and *PGK1* DMGs Discriminate PAH Patients vs. CTRLs

**Fig. 5:**
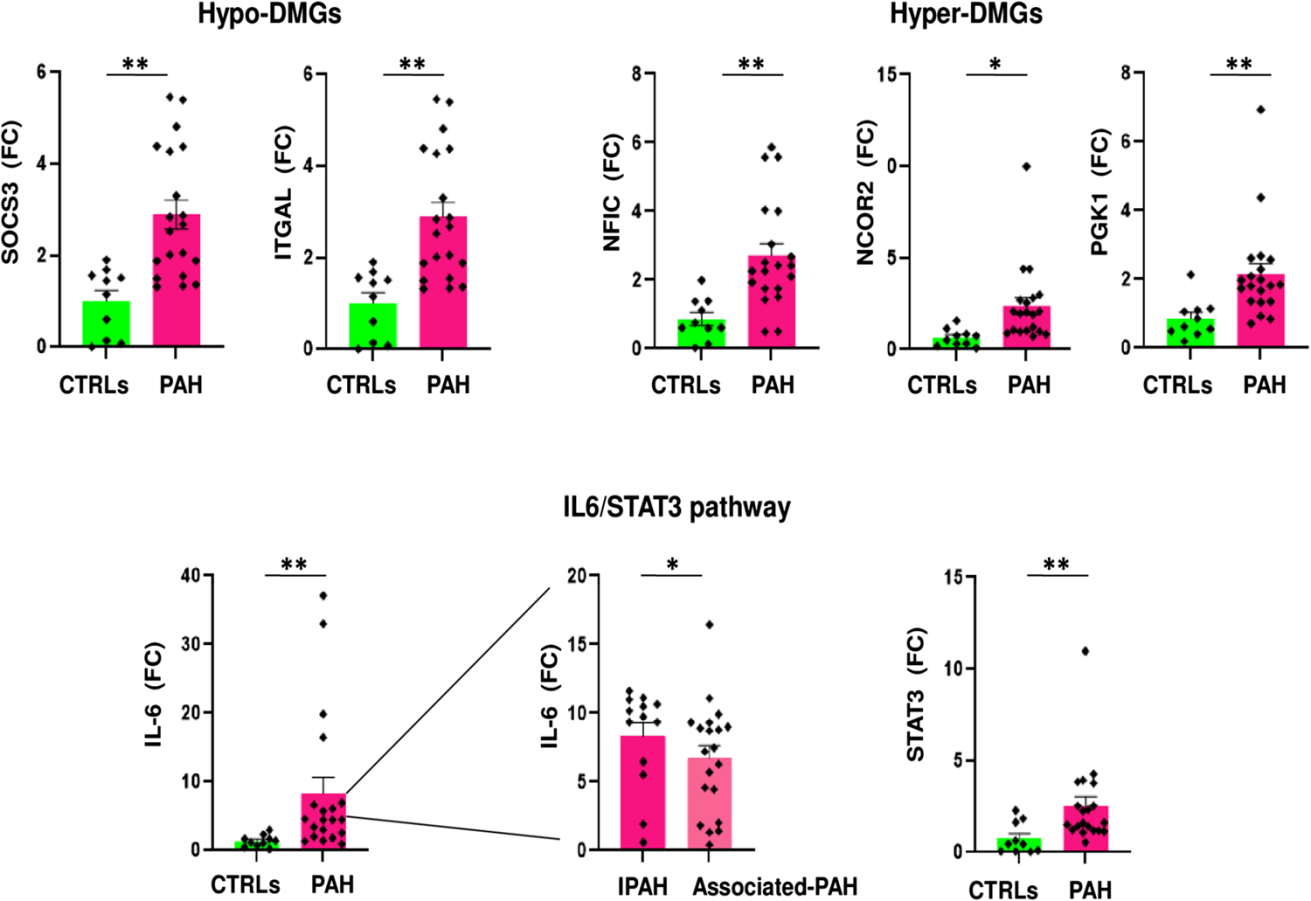
Network-oriented transcriptional profiles. Dot plots show the relative expression (FC) of hypomethylated and hypermethylated network-oriented DMGs and IL-6/STAT3 signaling axis in PBMCs from *N* = 20 PAH patients vs*. N* = 10 CTRLs (Wilcoxon rank sum exact test, *p < 0.05 vs. controls; **p < 0.01 vs*.* controls). Dot plots were performed using GraphPad Prism version 9.0.0 software. Abbreviations: ITGAL integrin subunit alpha L; GNAS, guanine nucleotide binding protein (G protein), alpha stimulating activity; GRM2, glutamate metabotropic receptor 2; LIMS2, LIM zinc finger domain containing 2; NCOR2, nuclear receptor corepressor 2; NFIC, nuclear factor I C; NR4A2, nuclear receptor subfamily 4 group A member 2; PAH, pulmonary arterial hypertension; PGK1, phosphoglycerate kinase 1; SOCS3, suppressor of cytokine signaling 3

Since promoter DNA methylation levels can affect gene expression at transcriptional level, we first determined if differences in mRNA levels of the 10 network-oriented DMGs were reliably detected in PBMCs by qRT-PCR. We chose PBMCs for two reasons: first, the recovery of CD4^+^ T cells from each peripheral blood biospecimen did not allow to extract also total mRNA; second, PBMCs represent a more easily accessible biospecimen than CD4^+^ T cells for potential clinical usefulness. For qRT-PCR experiments, we used PBMCs from *N* = 20 PAH patients (*N* = 10 Associated-PAH vs. 10 IPAH) vs. 10 CTRLs. In this experimental set, we included study participants from discovery set, validation set, as well as external subjects (Supplementary Table 13). Irrespective from promoter DNA methylation status, relative expression data (fold change, FC) showed a significant upregulation of *SOCS3* (Wilcoxon rank sum test, *p* ≤ *0.05*), *ITGAL* (Wilcoxon rank sum test, *p* ≤ *0.05*), *NFIC* (Wilcoxon rank sum test, *p* ≤ *0.05*), *NCOR2* (Wilcoxon rank sum test, *p* ≤ *0.05*), and *PGK1* (Wilcoxon rank sum test, *p* ≤ *0.05*) mRNA levels in PAH patients vs*.* CTRLs (Fig. 5). Since the network-oriented *SOCS3* gene is a negative feedback mediator of the IL-6 signaling [37–40], we investigated the transcriptional profiles of* IL-6*, IL-6 receptor (*IL-6R*), and signal transducer and activator of transcription 3 (*STAT3*) genes. The mRNA levels of the *IL-6* (Wilcoxon rank sum test, *p* ≤ 0.05) and *STAT3* (Wilcoxon rank sum test, *p* ≤ 0.05) genes were significantly upregulated in PAH patients vs. CTRLs. Among the transcriptional profiles evaluated in the PAH subnetwork, only the IL-6 upregulation significantly distinguished IPAH vs. Associated-PAH (Wilcoxon rank sum test, *p* ≤ 0.05) (Fig. 5) suggesting a potential phenotype-specific biomarker or drug target.

**Fig. 6:**
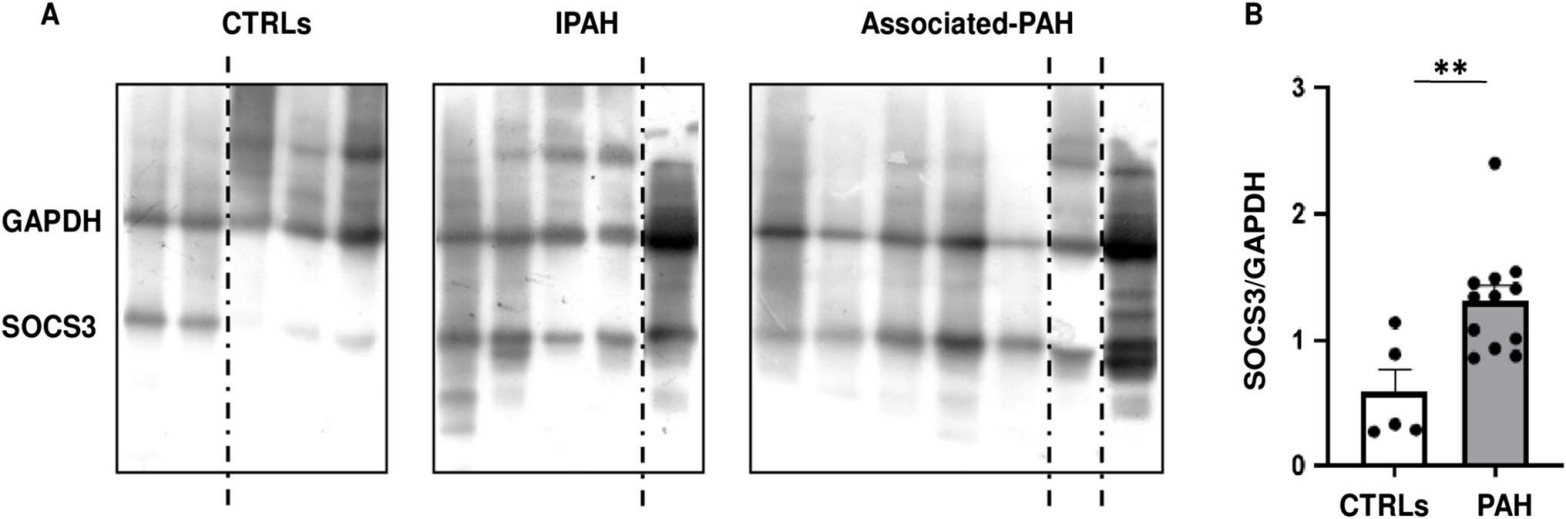
Network-oriented *SOCS3* expression profile. **A** Western blot analysis of SOCS3 protein expression in PBMCs isolated from CTRLs (*N* = 5) vs. IPAH (*N* = 5) and Associated-PAH (*N* = 7). The dashed black lines correspond to the cuts in the original film. **B** The dot plot shows the absolute values of the SOCS3/GAPDH ratio for each study participant. SOCS3 protein expression was significantly higher in PAH patients vs. CTRLs (two-tailed *t*-test; **p < 0.01). Dot plot was performed using GraphPad Prism version 9.0.0 software. Abbreviations: CTRLs, controls; GAPDH, glyceraldehyde-3-phosphate dehydrogenase; IPAH, idiopathic pulmonary arterial hypertension; SOCS3, suppressor of cytokine signaling 3

### Expression Profile of the Network-Oriented SOCS3 Protein Discriminates PAH Patients vs. CTRLs

We further focused our attention on the network-oriented *SOCS3* gene for two reasons: first, *SOCS3 *was the most recurrent gene among the top significant pathways into the PAH subnetwork (Fig. 3); second, *SOCS3* promoter hypomethylation was negatively associated with RAP and positively associated CI in both IPAH and Associated-PAH patients suggesting a biomarker of poor hemodynamics (Fig. 4B–C). Based on the availability and quality of PBMCs, we measured the expression of the SOCS3 protein by immunoblot in *N =* 5 CTRLs vs. *N* = 5 IPAH and *N* = 7 Associated-PAH patients (Fig. 6A and Supplementary Fig. 9). Clinical characteristics of PAH patients are described in Supplementary Table 14. According to *SOCS3 *promoter hypomethylation in CD4^+^ T cells and upregulation of mRNA levels in PBMCs (Fig. 5), we found that SOCS3 protein levels were significantly higher in PAH patients vs. CTRLs (Fig. 6B), with no difference between IPAH vs. Associated-PAH (two-tailed *t*-test; *p* < 0.05). This lets us to speculate that *SOCS3* might represent a hub gene in PAH pathogenesis and its progression.

## Discussion

The major findings of the present study are as follows: (1) circulating CD4^+^ T cell promoter methylation levels informed a network-based approach to identifying *SOCS3*, *GNAS*, *ITGAL*, *NCOR2*, *NFIC, NR4A2*, *GRM2*, *PGK1*, *STMN1*, and *LIMS2* genes as potentially involved in PAH pathogenesis or its progression, a finding that was validated by demonstrating that these genes strongly associated with RAP, CI, mPAP, or PVR; (2) mRNA levels of the network-oriented *SOCS3*, *ITGAL*, *NFIC*, *NCOR2*, and *PGK1* genes were significantly upregulated in PBMCs isolated from PAH patients vs. CTRLs; (3) of note, the *SOCS3* promoter hypomethylation correlated inversely with RAP and CI both in IPAH and Asssociated-PAH suggesting a potential prognostic biomarker; and (4) a significant *SOCS3* gene overexpression was confirmed at protein level in PBMCs from PAH patients vs. CTRLs supporting its role as hub gene in PAH. 

The goal of our study was to apply a novel network-oriented approach to patient-derived CD4^+^ T cell methylation profiles in order to identify potential PAH candidate genes and molecular pathways able to mirror clinical information. Interestingly, both in IPAH and Associated-PAH, *SOCS3* hypomethylation was negatively correlated with RAP and positively correlated with CI which are the most robust parameters of right ventricle function and prognosis in PAH. Despite the fact that association does not mean causation, this let us hypothesize that DNA methylation changes of the *SOCS3* gene might have a role in disease onset and its progression. Also, previous evidence reported an upregulation of *SOCS3* mRNA levels in fibroblast-activated macrophages accumulated in pulmonary arterioles from IPAH patients and a parallel upregulation of IL-6/STAT3 axis suggesting a potential drug target [31].

Promoter hypomethylation of the *ITGAL* gene in CD4^+^ T cells may contribute to its mRNA overexpression in PMBCs of patients with PAH vs. CTRLs. Also known as CD11a, the *ITGAL* gene encodes for the alpha L chain of integrins. The ITGAL protein is essential to inflammatory and immune responses by regulating adhesive and co-stimulatory interactions between CD4^+^ T cells and other cells. No evidence exists for involvement of the *ITGAL* gene in PAH; however, a prior study reported a parallel hypomethylation and mRNA overexpression of the *ITGAL* gene in circulating CD4^+^ T cells isolated from 18 patients with systemic sclerosis (of which only one had PAH) vs. 15 CTRLs leading to immunological abnormalities and fibrotic processes [41]. Thus, upregulation of the *ITGAL* gene may guide transmigration of CD4^+^ T cells and consequent infiltration into small pulmonary arterioles exacerbating vascular remodeling in PAH.

Despite the fact that *PGK1*, *NFIC*, and *NCOR2* gene promoters were hypermethylated in specific CpG sites, we found significantly increased mRNA levels in PBMCs of patients with PAH vs. CTRLs. This observation is in line with prior evidence showing a positive correlation between DNA methylation and gene expression in certain systems, thus countering the traditional dogma [42]. An explanation for this finding may be that DNA methylation is not itself alone sufficient to define the spectrum of gene expression but may only affect genes that are already silenced by other epigenetic-sensitive mechanisms [43]. In fact, the specific location of the methylation and its interaction with other epigenetic factors, particularly histone modifications and DNA-binding transcription factors, provide additional complexity to DNA methylation-dependent gene regulation processes [5].

A major strength of our study was the use of CD4^+^ T cells purified from peripheral blood specimens collected during RHC at first diagnosis (> 70%) or early follow-up (within 1 year). While it is true that lung tissues are necessary to potentially link methylation signatures to PAH pathogenesis, a great limitation is that lung biopsy is not usually performed in the routine management of PAH. Consequently, lung tissues are available only from end-stage PAH patients undergoing lung transplantation who were largely enrolled in previous omics-based analyses [1]. It is very difficult to clarify whether DNA methylation changes in lung tissues are really PAH-specific because the epigenome of end-stage patients might be merely a consequence of the overall compromised pulmonary status rather than a mirror of early drivers of disease.

Our interest on CD4^+^ T cells first arose from the evidence that pulmonary lesions in PAH patients are sites of a marked infiltration of CD4^+^ T cells [13–16]. In addition, CD4^+^ T cells with protective roles (Tregs) migrated to the lung in the early stages of PAH to suppress pulmonary inflammation [11], whereas Th2 cells contributed to muscularization of the pulmonary arterioles [12]. Our focus on circulating CD4^+^ T cells was particularly strengthened by prior data showing that the bone morphogenetic protein receptor type 2 (*BMPR2*) signaling axis, as a major driver of PAH pathogenesis [1], may contribute to thymic homeostasis by regulating commitment and differentiation of the T cell lineage [44]. Taken together, we considered reasonable and attractive the use of circulating CD4^+^ T cells to evaluate whether DNA methylation changes associated with hemodynamics, potentially informing the identification of readily accessible prognostic biomarkers.

Globally, our RRBS and subsequent network-oriented analysis showed that circulating CD4^+^ T cells were preferentially hypermethylated in PAH patients vs. CTRLs. This finding may be consistent with deleterious loss-of-function mutations in the ten-eleven translocation (TET) 2 demethylating enzyme found in Caucasian patients with IPAH associated with increased inflammation [6]. Previous studies suggested that promoter hypermethylation both in PBMCs and lung tissues was associated with *BMPR2* gene downregulation and progression of PAH [45, 46]. Interestingly, Bisserier et al. [46] demonstrated that an increased expression of the SIN3 transcription regulator family member A (SIN3A) repressor downregulated the levels of DNA methyltransferase 1 (DNMT1) while promoting the expression of the DNA demethylase TET1 in human pulmonary artery smooth muscle cells suggesting a potential epigenetic-based approach to attenuate PAH. Compared to previous studies, our genome-wide differential DNA methylation analysis did not identify specific dmCpGs annotated to the *BMPR2* promoter gene. The underlying reason can be that DNA methylation signatures are strongly dependent on cellular type as well as they can vary on the basis of sequencing platforms. We chose RRBS for the discovery analysis for two reasons: first, it is a preferable quantitative assay for revealing unusual methylated loci and minimizing DNA loss due to bisulfite-induced degradation when sample quantity is limited [47]; second, RRBS provides an enrichment in CGIs and promoter regions. We next performed the Infinium Human Methylation EPIC BeadChip analysis which employs oligonucleotide hybridization probes targeting CpG sites of interest in the validation cohort. Of note, the methylation trend for the network-oriented DMGs of the PAH subnetwork was the same in both next-generation sequencing platforms.

Our study presents some limitations. First, this is a pilot study aimed at exploring a novel research strategy which integrates DNA methylation, network analysis, and hemodynamics in PAH patients. The small size of both discovery and validation sets as well as their clinical heterogeneity do not allow to get a secure finding. Besides, the intrinsic nature of the epigenome-wide association studies do not allow to infer a cause-effect relationship of our PAH network-oriented candidate genes. Since diagnosis of PAH generally occurs in older patients, it was impossible to match PAH patients and blood donor volunteers for age, as described in another study [48]. However, no statistical difference in distribution of age for both CTRLs and PAH patients was found (Wilcoxon rank sum test, *p* = 0.07) (Data Supplement). Additionally, a linear regression analysis between the DNA methylation levels of each network-oriented DMGs and the age of both CTRLs and PAH patients revealed that the predictor variable (age) was not significantly related to the outcome variable (DMGs) for 8/10 network-oriented genes (Supplementary Fig. 10). According to a previous study [49], we can rule out the possibility that differential DNA methylation signatures between CTRLs and PAH patients may be due to the age. Furthermore, due to availability of biological biospecimens from our study population, we were not able to perform experimental validation (qRT-PCR and Western Blot) on CD4^+^ T cells but only in PBMC fraction. Larger prospective studies are needed to evaluate whether network-oriented DMGs may be used as potential biomarkers to optimize prognosis in PAH [1, 3, 50–52]. Nonetheless, circulating DNA methylome analyzed via network analysis may yield insight into early disease pathogenesis and suggest noninvasive biomarkers that can be used to optimize patient phenotyping and, possibly, predicted outcome in PAH.

## Supplementary Information

Below is the link to the electronic supplementary material.

Supplementary file1 (DOCX 2623 KB)

## Data Availability

All raw data obtained from RRBS and Infinium Human Methylation EPIC BeadChip platforms have been uploaded to the NCBI GEO database (accession ID: GSE165360).

## Abbreviations

BSG: Basigin
CGIs: CpG islands
CI: Cardiac index
CLEC4G: C-type lectin domain family 4 member G
CTBP2: C-terminal binding protein 2
DLC1: DLC1 Rho Gtpase activating protein
dmCpGs: Differentially methylated CpG sites
DMGs: Differentially methylated genes
DNMT3B: DNA methyltransferase 3 beta
DPPA4: Developmental pluripotency associated 4
gDNA: Genomic DNA
GNAS: Guanine nucleotide binding protein (G protein), alpha stimulating activity
GRM2: Glutamate metabotropic receptor 2
IL: Interleukin
ITGAL: Integrin subunit alpha L
LAMA5: Laminin subunit alpha 5
LIMS2: LIM zinc finger domain containing 2
mPAP: Mean pulmonary arterial pressure
NCOR2: Nuclear receptor corepressor 2
NFIC: Nuclear factor I C
NR4A2: Nuclear receptor subfamily 4 group A member 2
PAH: Pulmonary arterial hypertension
PCWP: Pulmonary capillary wedge pressure
PBMCs: Peripheral blood mononuclear cells
PGK1: Phosphoglycerate kinase 1
PKP1: Plakophilin 1
PPIs: Protein-protein interactions
PVR: Pulmonary vascular pressure
qRT-PCR: Quantitative real-time PCR assay
RAP: Right atrial pressure
RHC: Right heart catheterization
RNF166: Ring finger protein 166
RPTOR: Regulatory associated protein of MTOR complex 1
RRBS: Reduced representation bisulfite sequencing
SF3A1: Splicing factor 3a subunit 1
SH3BP4: SH3 domain binding protein 4
SOCS3: Suppressor of cytokine signaling 3
STAT3: Signal transducer and activator of transcription 3
STMN1: Stathmin 1
TSS: Transcription start site
UTRs: Untranslated regions
