# Supplementary material for: Association Between Circulating CD4+ T Cell Methylation Signatures of Network-Oriented SOCS3 Gene and Hemodynamics in Patients Suffering Pulmonary Arterial Hypertension
Source: J Cardiovasc Transl Res. 2022 Aug 12;16(1):17–30. doi: 10.1007/s12265-022-10294-1 (PMC9944731; doi:10.1007/s12265-022-10294-1)
Supplement: Supplementary file 1 — Supplementary file1 (DOCX 2623 KB) [file 12265_2022_10294_MOESM1_ESM.docx]

**Association between circulating CD4^+^ T cell methylation signatures of network-oriented SOCS3 gene and hemodynamics in patients suffering pulmonary arterial hypertension**

Giuditta Benincasa^a^*#, PhD, Bradley A Maron^b^*, MD, Ornella Affinito^c^*, PhD, Michele D’Alto^d^, MD, PhD, Monica Franzese^c^, PhD, Paola Argiento^d^, MD, PhD, Concetta Schiano^a^, PhD, Emanuele Romeo^d^, MD, PhD, Paola Bontempo^e^, MD, PhD, Paolo Golino^d^, MD, PhD, LiberatoBerrino^e^, MD, PhD, Joseph Loscalzo^b^, MD, PhD, Claudio Napoli^a,c^, MD, PhD

^1^Department of Advanced Medical and Surgical Sciences (DAMSS), University of Campania "Luigi Vanvitelli", 80138 Naples, Italy.

^2^Division of Cardiovascular Medicine, Department of Medicine, Brigham and Women's Hospital, and Harvard Medical School.

^3^IRCCS SDN, Naples, Italy.

^4^Department of Cardiology, Monaldi Hospital, University of Campania "Luigi Vanvitelli", Naples, Italy.

^5^Department of Precision Medicine, University of Campania "Luigi Vanvitelli", Naples, Italy.

^6^Department of Experimental Medicine, University of Campania “Luigi Vanvitelli”, Naples, Italy.

^*^These Authors equally contributed

**^#^Address for Correspondence:**

Email: [giuditta.benincasa@unicampania.it](mailto:giuditta.benincasa@unicampania.it);

Phone: +390815667916 and Fax: +390815665071.

**EXPANDED MATERIALS AND METHODS**

**Collection of PBMCs, isolation of CD4^+^ T cells, and extraction of genomic DNA (gDNA)**

From each study participant (N=25 PAH patients and N=16 CTRLs), peripheral blood samples were collected in EDTA tubes and centrifuged at 1900 x g for 10 minutes at room temperature within 2 hours of collection to separate plasma and cellular components. Peripheral blood samples were diluted with 30 mL of PBS, and the leukocyte ring was stratified by density centrifugation on a Ficoll gradient using Histopaque®-1077 (800 g, 20 minutes, at room temperature). PBMCs were next collected and washed (2x) in 50 mL PBS (1600 g, 5 minutes, at room temperature) and resuspended in 3 mL PBS. Two mL of cellular suspension were pipetted in a glass tube to purify CD4^+^ T cells using the EasySep™ Human CD4^+^ T Cell Isolation Kit (Stem Cell), according to the manufacturer’s instructions. This assay was based on a negative selection procedure for which antibodies targeting specific surface markers of non-CD4^+^ T cells coupled with magnetic particles removed unwanted cells. gDNA samples from CD4^+^ T cells were immediately extracted using DNeasy Blood & Tissue Kit (Qiagen), according to manufacturer’s instructions. Both concentration and purity of gDNA were determined using a NanoDrop spectrophotometer (Thermo Scientific) with A260/A280 and A260/A230 as standard absorbance ratios. The integrity of all gDNA samples was checked on 1% agarose gel, and the samples stored at -80^o^C before sequencing. Based on recovery of PBMCs for each study participant, some cellular aliquots were stored in TRIZOL reagent at -80^o^C until further qRT-PCR experiments and/or without solvent at -80^o^C until further Western Blot experiments.

**Genome-wide DNA methylation analysis**

***Discovery set-RRBS****.*Briefly, approximately 2,5-3.0 μg of each gDNA sample was digested with the MspI restriction enzyme and purified with the GeneJet PCR Purification Kit (Thermo Fisher Scientific) (Supplemental Appendix).All libraries were prepared by TruSeq Library Prep Kit (Illumina), and bisulfite conversion was produced using the EZ DNA Methylation-Gold Kit (ZymoResearch). A DNA amplification reaction was performed using the PfuTurboCxHotstart DNA Polymerase kit (Agilent Technologies, USA), and the amplified fragments were purified by AMPure XP Beads. Finally, the fragments were quantified using the Agilent 4200 TapeStation (Agilent Technologies). Each DNA library was analyzed by a paired-end sequencing read (2×75 cycles) on Illumina Nextseq 500.

***Validation set-Infinium Human MethylationEPICBeadChip****.* Briefly, 350-500 ng of gDNA from each sample was bisulfite converted using the EZ-96 DNA Methylation Kit (Zymo Research Corporation, Orange, US), according to the manufacturer’s recommendations. Each sample was whole genome-amplified and enzymatically fragmented following the instructions in the Illumina Infinium HD Assay Methylation Protocol guide. Bisulfite-converted DNA was amplified, fragmented, and hybridized to the Illumina Infinium Human MethylationEPICBeadchip using the standard Illumina protocol.

**DNA Sequence processing and alignment**

***Discovery-RRBS****.* RRBS raw reads were assessed for quality using FastQC (v011.8, Babraham Bioinformatics, UK) and trimmed to remove Illumina adaptors and low-quality reads using TrimGalore (v0.6.3, Babraham Bioinformatics, UK) with the default settings. Trimming was performed using the *rrbs* parameter in order to trim the first 2 bases from the 3’ end of the sequence, artificially added during RRBS library preparation. Trimmed reads were then mapped to the *in silico*bisulfite-converted human reference genome (GRCh38) using Bismark v.0.22.1^1^ with default parameters, the aligner Bowtie2 (v2.3.5.1)^2^, and a *non-directional* parameter applied. Only the reads that were uniquely mapped were used to make cytosine methylation calls using the *Bismark methylation extractor.* On average, across the 14 samples investigated for CD4^+^ T cells, 21,284,371 reads were obtained, 21,144,905 reads were retained after quality filtering, and 12,859,132 reads were uniquely mapped to the reference genome with 60% mapping efficiency and the proportion of C methylated in a CpG context of approximately 39%. Only methylation within the CpG context was considered for further analysis. To prevent PCR bias and to increase the power of the statistical test, CpG sites covering less than 10 reads or more than the 99.9^th^ percentile of coverage distribution in each sample were filtered out.

***Validation-Infinium Human Methylation EPICBeadChip****.* Microarrays were scanned using the Illumina iScan-Scanner. Data were quantile-normalized for red and green channels separately using the function normalize.quantiles from the Bioconductor package "preprocessCore." DNA methylation values, described as beta values, are recorded for each locus in each sample. The beta value difference has been calculated using the mean beta values of the two groups. DNA methylation beta values are continuous variables between 0 and 1, representing the percentage methylation of a given cytosine corresponding to the ratio of the methylated signal over the sum of the methylated and unmethylated signals. For differential methylation analysis, the difference of beta values has been calculated using the mean beta values of the two groups.

***Transcriptional profiles (qRT-PCR)***

RNA quantity and quality were determined using a NanoDrop ND-1000 spectrophotometer (Thermo Fischer Scientific, USA). RNA (500ng) was reverse transcribed with SuperScript® III First-Strand Synthesis System for RT-PCR (Thermo Fischer Scientific, USA), according to the manufacturer’s instructions, in 20 μL reaction. The relative expression levels of mRNA were measured with a CFX96 Touch Real-Time PCR Detection System (BioRad Laboratories, Ltd, USA) using iQ™ SYBR® Green Supermix (BioRad Laboratories, Ltd, USA) and 300 nM of each primer pair. Primers were designed by Primer 3 software (<http://bioinfo.ut.ee/primer3-0.4.0/>) and synthesized by Life Technologies. The specificity of each oligonucleotide pair was verified with the BLAST program. Melt curve analysis was performed to verify a single product species.

***Protein profiles****(****Western Blot)***

Proteins were obtained after suspension of PBMCs in lysis buffer (50 mM Tris-HCl pH 7.4, 150 mM NaCl, 1% NP40, 10 mM NaF, 1 mM PMSF, and protease inhibitor cocktail), for 15 min at 4°C. After centrifugation, protein concentration was determined by Bradford assay (Bio-Rad #5000006). A total of 30 μg of proteins was loaded on 12% sodium dodecyl sulfate polyacrylamide gel (SDS-PAGE), and transferred to nitrocellulose membrane membranes over-night. After a step of blocking in 5% milk in Tris-buffered saline with Tween (TBST; 10 mM Tris pH 8.0, 150 mM NaCl, 0.5% Tween 20), the membranes were washed and incubated with the mouse anti-human anti-SOCS3 (OriGene, #TA502991) at a dilution 1:1,000 overnight at 4°C. Detection was performed with an enhanced chemiluminescence system (Amersham Biosciences) according to the manufacturer's protocol. Densitometry was performed using ImageJ software^3^ and the gene of interest was normalized to the glyceraldehyde 3-phosphate dehydrogenase (*GAPDH*) gene as loading control.

**Demographic and clinical characteristics of PAH patents *vs.* CTRLs enrolled in each experimental set**

***RRBS-Discovery Set****.* In our discovery population, we enrolled N=7 PAH patients (5 F and 2 M) *vs.* N=7 CTRLs (4 F and 3 M) (**SupplementaryTable 4**). PAH patients consisted of N=4 idiopathic PAH (IPAH) and N=3 Associated-PAH of which N=2 were associated with systemic sclerosis (PAH-SSc) and N=1 with portal hypertension (POPH). Most of PAH patients were enrolled at first diagnosis (71.42%) whereas a minor percentage (28.58%) at early follow-up. The mean age was 57.7 ± 16.3 years in PAH patients *vs.* 42.2 ± 10.0 years in CTRLs. No statistical difference in distribution of age for the two groups (CTRLs and PAH) was found (Wilcoxon rank sum test, p-value = 0.07301). The functional class of PAH patients was New York Heart Association (NYHA) Class II (N=4, 57.14%) and III (N=3, 42.86%). The mean right atrial pressure (RAP) was 5.7 ± 3.04 mmHg; the mean pulmonary arterial pressure (mPAP) was 31.1 ± 4.88 mmHg; the mean pulmonary capillary wedge pressure (PCWP) was 9.86 ± 2.6 mmHg; the mean pulmonary vascular resistance (PVR) was 5.52 ± 2.34 Wood Units; and the mean cardiac index (CI) was 2.73± 0.94 L/min x m2. Risk stratification evaluation classified patients at low risk (N=3, 42.9%), and high risk (N=4, 57.1 %%).

***EPIC-Validation Set****.* We enrolled 5 PAH patients (3 F and 2 M) consisting of IPAH (N=3), PAH associated with systemic sclerosis (PAH-SSc, N=1), and PAH associated with congenital heart disease (PAH-CHD, N=1) (**SupplementaryTable 11**). PAH patients were compared to N=4 CTRLs (2 F and 2 M). The mean age was 71 ± 5 years in PAH patients *vs.*28.2 ± 3.9 years in CTRLs. The functional class of PAH patients was in NYHA Class II (N=2, 40%) and III (N=3, 60%). PAH patients were at first diagnosis (40%) or follow-up (60%). The mean RAP was 4.60 ± 3.71 mmHg; the mean mPAP was 39.0 ± 13.5mmHg; the mean PAWP was 9.80 ±0.83 mmHg; the mean PVR was 8.78 ± 5.82 Wood units; and the mean CI was 2.04 ± 0.35 L/min x m2. Risk stratification evaluation classified patients at intermediate risk (N=2, 40%) and high risk (N=3, 60%).

**qRT-PCR.** We enrolled 20 PAH patients (10 F and 10 M) consisting of IPAH (N=10), PAH associated with systemic sclerosis (PAH-SSc, N=4), PAH associated with congenital heart disease (PAH-CHD, N=4), and PAH associated with portal hypertension (N=2) (**SupplementaryTable 13**). PAH patients were compared to N=10 CTRLs (6 F and 4 M). The mean age was 63.8.4 ± 14.5 years in PAH patients *vs.* 46.4 ± 11.8 years in CTRLs. The functional class of PAH patients was in NYHA Class I (N=1, 5%), II (N=9, 45%), and III (N=10, 50%). The mean RAP was 5.10 ± 3.71 mmHg; the mean mPAP was 36.4 ± 12.5mmHg; the mean PAWP was 9.15 ± 2.54mmHg; the mean PVR was 6.44 ± 4.61 Wood Units; and the mean CI was 2.77 ± 0.79 L/min x m2. Risk stratification evaluation classified patients at low risk (N=9, 45%), intermediate risk (N=4, 20%), and high risk (N=7, 35%).

**Western Blotting.** We enrolled 12 patients with PAH (7 F and 5 M) consisting of IPAH (N=5), PAH associated with systemic sclerosis (PAH-SSc, N=4), PAH associated with portal hypertension (POPH, N=2), and PAH associated with congenital heart disease (PAH-CHD, N=1) (**SupplementaryTable 14**). PAH patients were compared to N=5 CTRLs (2 M and 3 F) The mean age was 62.9 ± 10.0 years in PAH patients *vs.*46.4 ± 13.9 years in CTRLs. The functional class of PAH patients was in NYHA Class I (N=1, 8.3%), II (N=6, 50%) and III (N=5, 41.7%). The mean RAP was 7.58 ± 8.16mmHg; the mean mPAP was 35.3 ± 9.27mmHg; the mean PCWP was 9.7 ± 3.02 mmHg; the mean PVR was 4.88 ± 2.65 Wood Units; and the mean CI was 3. 08 ± 1.0 L/min x m2. Risk stratification evaluation classified patients at low risk (N=6, 50%), intermediate risk (N=1, 8.3%), and high risk (N=5, 41.7%).

**References**

1. Krueger F, Andrews SR. Bismark: a flexible aligner and methylation caller for Bisulfite-Seq applications. Bioinformatics. 2011;27:1571-1572. doi: 10.1093/bioinformatics/btr167.

2. Langmead B, Salzberg SL. Fast gapped-read alignment with Bowtie 2. Nat Methods. 2012;9:357. doi: 10.1038/nmeth.1923.

3. Schneider CA, Rasband WS, Eliceiri KW. NIH Image to ImageJ: 25 years of image analysis. Nat Methods. 2012;9:671-675. doi: 10.1038/nmeth.2089.

**Supplementary Table 1.** Clinical characteristics of CLEOPAHTRA study population

| **Variable** | **PAH (N=25)** | **CTRLs (N=16)** |
| --- | --- | --- |
| **Age, y** | 62.2 ± 14.8 | 41.62± 12.29 |
| **Female** | 18 (72.0) | 9 (56.0) |
| **PAH phenotype** |  |  |
| **IPAH** | 13 (52.0) |  |
| **PAH-CHD** | 5 (20.0) |  |
| **PAH-SSc** | 5 (20.0) |  |
| **POPH** | 2 (8.0) |  |
| **NYHA class** |  |  |
| **I** | 1 (4.0) |  |
| **II** | 13 (52.0) |  |
| **III** | 11 (44.0) |  |
| **Invasive hemodynamics** |  |  |
| **RAP, mmHg** | 6.36 ± 6.45 |  |
| **mPAP, mmHg** | 35.6 ± 11.8 |  |
| **PCWP, mmHg** | 9.56 ± 2.6 |  |
| **PVR, WU** | 5.52 ± 2.55 |  |
| **CI, L/min/m2** | 3. 0 ± 0.88 |  |
| **Risk category** |  |  |
| **Low-risk** | 10 (40.0) |  |
| **Intermediate-risk** | 10 (40.0) |  |
| **High-risk** | 5 (20.0) |  |

Data are n (%) or mean ± standard deviation (SD). Abbreviations: CI: Cardiac Index; CTRLs: Healthy Control; IPAH: Idiopathic PAH; mPAP: Mean Pulmonary Arterial Pressure; NYHA: New York Heart Association; PAH-CHD: PAH Associated with Congenital Heart Disease; PAH-SSc: PAH Associated with Systemic Sclerosis: PCWP: Pulmonary Capillary Wedge Pressure; POPH: PAH Associated with Portal Hypertension; PVR: Pulmonary Vascular Resistance.

**Supplementary Table 2**. Statistics of libraries per sample

| **Sample** | **# Reads (FastQ)** | **# Reads (FastQ_Trim)** | **% Alignment** | **Unique Hits** | **# reads NOT aligned** | **# reads NOT map uniquely** | **% Meth(mC in CpG context)** | **%**  **Meth**  **(mC in CHG context)** | **%**  **Meth**  **(mC in CHH context)** | **Total number of C** | **Total number of methylated C’s in CpG context** | **Total number of unmethylated C's in CpG context** | **Sum C's in CpG context** |
| --- | --- | --- | --- | --- | --- | --- | --- | --- | --- | --- | --- | --- | --- |
| **PAH 1** | **R1: 14061616** | **R1: 13884779** | **67.3%** | **9345322** | **2533951** | **2005506** | **48.3%** | **0.8%** | **0.7%** | **258255996** | **18128148** | **19433441** | **37561589** |
|  | **R2: 14061616** | **R2: 13884779** |  |  |  |  |  |  |  |  |  |  |  |
| **PAH 2** | **R1: 14326772** | **R1: 14164286** | **63.1%** | **8932203** | **2549154** | **2682929** | **43.0%** | **0.9%** | **0.8%** | **227126853** | **15921277** | **21098016** | **37019293** |
|  | **R2: 14326772** | **R2: 14164286** |  |  |  |  |  |  |  |  |  |  |  |
| **PAH 3** | **R1: 19459942** | **R1: 19246518** | **63.2%** | **12155578** | **3159163** | **3931777** | **39.6%** | **0.7%** | **0.5%** | **297610685** | **20137677** | **30752741** | **50890418** |
|  | **R2: 19459942** | **R2: 19246518** |  |  |  |  |  |  |  |  |  |  |  |
| **PAH 4** | **R1: 24925939** | **R1: 24628806** | **56.4%** | **13887334** | **6201050** | **4540422** | **42.5%** | **1.2%** | **1.0%** | **343229332** | **23532746** | **31792268** | **55325014** |
|  | **R2: 24925939** | **R2: 24628806** |  |  |  |  |  |  |  |  |  |  |  |
| **PAH 5** | **R1: 21518739** | **R1: 21232996** | **57.4%** | **12182206** | **5829474** | **3221316** | **42.8%** | **1.4%** | **1.3%** | **315645021** | **21187570** | **28339544** | **49527114** |
|  | **R2: 21518739** | **R2: 21232996** |  |  |  |  |  |  |  |  |  |  |  |
| **PAH 6** | **R1: 29143100** | **R1: 29051196** | **63.8%** | **18526526** | **3977265** | **6547405** | **37.9%** | **0.4%** | **0.2%** | **434982045** | **28933041** | **47378791** | **76311832** |
|  | **R2: 29143100** | **R2: 29051196** |  |  |  |  |  |  |  |  |  |  |  |
| **PAH 7** | **R1: 21138741** | **R1: 21079098** | **62.5%** | **13179210** | **2969666** | **4930222** | **40.0%** | **0.5%** | **0.4%** | **310813696** | **21388529** | **32092383** | **53480912** |
|  | **R2: 21138741** | **R2: 21079098** |  |  |  |  |  |  |  |  |  |  |  |
| **CTRL 1** | **R1: 17487788** | **R1: 17381744** | **55.7%** | **9688986** | **5119155** | **2573603** | **39.8%** | **1.0%** | **0.9%** | **238857956** | **16214273** | **24532948** | **40747221** |
|  | **R2: 17487788** | **R2: 17381744** |  |  |  |  |  |  |  |  |  |  |  |
| **CTRL 2** | **R1: 18112858** | **R1: 17995487** | **55.4%** | **9977841** | **4516149** | **3501497** | **31.0%** | **0.5%** | **0.3%** | **239528724** | **14410717** | **32010008** | **46420725** |
|  | **R2: 18112858** | **R2: 17995487** |  |  |  |  |  |  |  |  |  |  |  |
| **CTRL 3** | **R1: 18938892** | **R1: 18815868** | **56.0%** | **10539042** | **4775594** | **3501232** | **33.9%** | **0.6%** | **0.4%** | **253102025** | **15963665** | **31160764** | **47124429** |
|  | **R2: 18938892** | **R2: 18815868** |  |  |  |  |  |  |  |  |  |  |  |
| **CTRL 4** | **R1: 23218204** | **R1: 23141147** | **61.9%** | **14322120** | **3422483** | **5396544** | **38.7%** | **0.5%** | **0.3%** | **337315649** | **22591073** | **35819614** | **58410687** |
|  | **R2: 23218204** | **R2: 23141147** |  |  |  |  |  |  |  |  |  |  |  |
| **CTRL 5** | **R1: 24138948** | **R1: 24064148** | **63.2%** | **15205575** | **3312695** | **5545878** | **39.7%** | **0.6%** | **0.4%** | **349921685** | **23791104** | **36098286** | **59889390** |
|  | **R2: 24138948** | **R2: 24064148** |  |  |  |  |  |  |  |  |  |  |  |
| **CTRL 6** | **R1: 25331407** | **R1: 25242892** | **63.4%** | **15992661** | **3505015** | **5745216** | **34.3%** | **0.4%** | **0.2%** | **371255888** | **23176543** | **44379497** | **67556040** |
|  | **R2: 25331407** | **R2: 25242892** |  |  |  |  |  |  |  |  |  |  |  |
| **CTRL 7** | **R1: 26178249** | **R1: 26099707** | **61.7%** | **16093237** | **3555007** | **6451463** | **36.0%** | **0.4%** | **0.2%** | **364260320** | **23499526** | **41764813** | **65264339** |
|  | **R2: 26178249** | **R2: 26099707** |  |  |  |  |  |  |  |  |  |  |  |
| **Mean** |  |  |  |  |  |  |  |  |  |  |  |  |  |
|  | **21284371,07** | **21144905,14** | **60,78571429** | **12859131,5** | **3958987,214** | **4326786,429** | **39,10714286** | **0,7071428571** | **0,5428571429** | **310136133,9** | **20633992,07** | **32618079,57** | **53252071,64** |
|  | **21284371** | **21144905** | **61** | **12859132** | **3958987** | **4326786** | **39** | **1** | **1** | **310136134** | **20633992** | **32618080** | **53252072** |

*Abbreviations: CTRL:* Healthy Control;PAH: Pulmonary Arterial Hypertension.

**Supplementary Table 3**.Reverse and forward Primers for qRT-PCR experiments

| **Gene** | **Chr position** | **Forward** | **Reverse** | **Product size (bp)** | **Tm (°C)** |
| --- | --- | --- | --- | --- | --- |
| ***SOCS3*** | chr17 | CCCCAGAAGAGCCTATTACATC | CAGCTGGGTGACTTTCTCAT | 151 | 60 |
| ***GNAS*** | chr20 | TTCCAGAATTTGCTCGCTACA | GGATGTTCTCAGTGTCCACAG | 183 | 60 |
| ***ITGAL*** | chr16 | AAGTCAAGCACATGTACCAGG | GCCTCTCCAGATCCTCATAGT | 174 | 62 |
| ***NFIC*** | chr19 | GACAAGTCACCATTCAACAGC | GACAAGATCTTTGAGCGGGTC | 240 | 62 |
| ***NCOR2*** | chr12 | AGCGCACCTATGACATGATG | GATCCCTTGTGTGATGGACC | 155 | 60 |
| ***NR4A2*** | chr2 | TGGTTCGCACAGACAGTTTAA | AGAATTGCTGGATATGCTGGG | 225 | 60 |
| ***GRM2*** | chr3 | TGAGCAGGAGTCCAAGATCA | TTGTAGCGGCCAATACCATC | 246 | 60 |
| ***PGK1*** | chrX | GGGGTATTTGAATGGGAAGCT | GCTGACTTTATCCTCCGTGTT | 150 | 62 |
| ***STMN1*** | chr1 | GCTGACTTTATCCTCCGTGTT | TGTTCTTCCGCACTTCTTCAA | 221 | 60 |
| ***LIMS2*** | chr2 | CACTATGAGAAGAAGGGCCTG | CTTGTTCTTCAGGGTGAGCTT | 180 | 62 |
| ***IL-6*** | chr7 | ACATGTGTGAAAGCAGCAAAG | CTGGCTTGTTCCTCACTACTC | 196 | 60 |
| ***IL-6R*** | chr1 | CCTTTCAGGGTTGTGGAATCT | CGATATCTGAGCTCAAACCGT | 154 | 62 |
| ***STAT3*** | chr17 | CGATGGAGTACGTGCAGAAA | TGTTGACGGGTCTGAAGTTG | 160 | 60 |
| ***RPS18*** | chr2 | CGATGGGCGGCGGAAAATA | CTGCTTTCCTCAACACCACA | 86 | 60 |

*Abbreviations:* Bp: Base Pair*; ITGAL*: Integrin Subunit Alpha L; *GNAS*: Guanine Nucleotide Binding Protein (G Protein), Alpha Stimulating Activity; *GRM2*: Glutamate Metabotropic Receptor 2; *IL-6*: Interleukin-6; *IL-6R*: Interleukin-6 Receptor; *LIMS2*: LIM Zinc Finger Domain Containing 2; *NCOR2*: Nuclear Receptor Corepressor 2; *NFIC*: Nuclear Factor I C; *NR4A2*: Nuclear Receptor Subfamily 4 Group A Member 2; PAH: Pulmonary Arterial Hypertension; *PGK1*: Phosphoglycerate Kinase 1; qRT-PCR: Quantitative Real Time Polymerase Chain Reaction; *RPS18*: Ribosomal Protein S18; *STAT3*: Signal Transducer and Activator of Transcription 3; *SOCS3*: Suppressor of Cytokine Signaling 3; *STMN1*: Stathmin.

**Supplementary Table 4**.Characteristics of discovery set (RRBS)

| **Variable** | **PAH (N=7)** | **CTRLs (N=7)** |
| --- | --- | --- |
| **Age, y** | 57.7± 16.3 | 42.2± 10.0 |
| **Female** | 5 (71.4) | 4 (57.1) |
| **PAH phenotype** |  |  |
| **IPAH** | 4 (57.1) |  |
| **PAH-SSc** | 2 (28.6) |  |
| **POPH** | 1 (14.3) |  |
| **NYHA class** |  |  |
| **II** | 4 (57.14) |  |
| **III** | 3 (42.86) |  |
| **Time of biospecimen collection** |  |  |
| **First diagnosis** | 5 (71.42) |  |
| **Follow-up**  (within 1 year from diagnosis) | 2 (28.58) |  |
| **Invasive hemodynamics** |  |  |
| **RAP, mmHg** | 5.71± 3.04 |  |
| **mPAP, mmHg** | 31.1± 4.88 |  |
| **PCWP, mmHg** | 9.86± 2.6 |  |
| **PVR, WU** | 5.52 ± 2.34 |  |
| **CI, L/min/m2** | 2.73± 0.94 |  |
| **Risk category** |  |  |
| **Low-risk** | 3 (42.9) |  |
| **High-risk** | 4 (57.1) |  |

Data are n (%) or mean ± standard deviation (SD). *Abbreviations:* CI: Cardiac Index; CTRL: Healthy Control; IPAH: Idiopathic PAH; mPAP: Mean Pulmonary Arterial Pressure; NYHA: New York Heart Association; PAH-SSc: PAH Associated with Systemic Sclerosis: PCWP: Pulmonary Capillary Wedge Pressure; POPH: PAH Associated with Portal Hypertension; PVR: Pulmonary Vascular Resistance; RAP: Right Atrial Pressure; RRBS: Reduced Representation Bisulfite Sequencing

**Supplementary Table 5**. Top 40 hypermethylated dmCpGs in PAH patients *vs.* CTRLs

| **Coordinates_dmCpGs** | **p.value** | **q.value** | **Meth.diff** | **Genomic Context** | **Gene Distance**  **to TSS** | **Gene**  **Nearest** | **Gene Name** | **CGIs** | **Shores** | **Other** |
| --- | --- | --- | --- | --- | --- | --- | --- | --- | --- | --- |
| **chrX_135436004_135436004** | **1,74E-14** | **4,17E-10** | **44,45244** | **Exon** | **14061** | **SMIM10L2A** | **small integral membrane protein 10 like 2A** | **0** | **1** | **0** |
| **chr21_39396345_39396345** | **6,49E-13** | **9,18E-09** | **43,43434** | **Intron** | **3449** | **WRB** | **tryptophanrichbasicprotein** | **0** | **0** | **1** |
| **chr9_101719185_101719185** | **1,38E-13** | **2,59E-09** | **41,09859** | **Intron** | **19395** | **GRIN3A** | **glutamateionotropic receptor NMDA typesubunit 3A** | **0** | **0** | **1** |
| **chr5_180313743_180313743** | **2,31E-17** | **1,20E-12** | **40,70298** | **Intron** | **10621** | **GFPT2** | **glutamine-fructose-6-phosphate transaminase 2** | **1** | **0** | **0** |
| **chr22_11600409_11600409** | **7,31E-20** | **1,14E-14** | **40,56415** | **DistalIntergenic** | **-639071** | **FRG1FP** | **FSHD region gene 1 family member F, pseudogene** | **0** | **1** | **0** |
| **chr7_158128733_158128733** | **3,17E-11** | **2,29E-07** | **39,26282** | **Intron** | **76544** | **PTPRN2** | **protein tyrosine phosphatase receptor type N2** | **0** | **0** | **1** |
| **chr1_34843655_34843655** | **4,74E-10** | **2,20E-06** | **38,40144** | **Intron** | **15390** | **SMIM12** | **small integral membrane protein 12** | **0** | **0** | **1** |
| **chr19_54457896_54457896** | **1,16E-12** | **1,39E-08** | **38,21378** | **Promoter (<=1kb)** | **-519** | **LENG8** | **leukocyte receptor cluster member 8** | **0** | **1** | **0** |
| **chr7_70760226_70760226** | **1,72E-10** | **9,48E-07** | **38,07918** | **Intron** | **-3058** | **AUTS2** | **activator of transcription and developmental regulator AUTS2** | **0** | **0** | **1** |
| **chr22_11628721_11628721** | **1,93E-18** | **1,71E-13** | **37,42034** | **DistalIntergenic** | **-667383** | **FRG1FP** | **FSHD region gene 1 family member F, pseudogene** | **1** | **0** | **0** |
| **chr13_28486496_28486496** | **1,18E-19** | **1,47E-14** | **36,96231** | **Intron** | **8583** | **FLT1** | **fmsrelatedtyrosinekinase 1** | **0** | **0** | **1** |
| **chr3_127227122_127227122** | **8,46E-13** | **1,12E-08** | **36,71469** | **DistalIntergenic** | **33991** | **C3orf56** | **chromosome 3 open reading frame 56** | **0** | **0** | **1** |
| **chr1_5651369_5651369** | **2,90E-13** | **4,63E-09** | **36,70886** | **Intron** | **211372** | **MIR4689** | **microRNA 4689** | **0** | **0** | **1** |
| **chr5_180313824_180313824** | **4,92E-14** | **9,87E-10** | **36,56085** | **Exon** | **10540** | **GFPT2** | **glutamine-fructose-6-phosphate transaminase 2** | **1** | **0** | **0** |
| **chr22_11628722_11628722** | **7,65E-24** | **2,38E-18** | **36,53808** | **DistalIntergenic** | **-667384** | **FRG1FP** | **FSHD region gene 1 family member F, pseudogene** | **1** | **0** | **0** |
| **chrX_324304_324304** | **1,08E-12** | **1,32E-08** | **35,68237** | **DistalIntergenic** | **-5485** | **GTPBP6** | **GTP bindingprotein 6 (putative)** | **1** | **0** | **0** |
| **chr9_122226147_122226147** | **3,50E-15** | **1,08E-10** | **35,05342** | **Promoter (<=1kb)** | **614** | **LHX6** | **LIM homeobox 6** | **1** | **0** | **0** |
| **chr22_36548974_36548974** | **2,59E-12** | **2,80E-08** | **34,98614** | **DistalIntergenic** | **-19538** | **EIF3D** | **eukaryotic translation initiation factor 3 subunit D** | **0** | **0** | **1** |
| **chr5_180313765_180313765** | **8,77E-13** | **1,14E-08** | **34,81126** | **Intron** | **10599** | **GFPT2** | **glutamine-fructose-6-phosphate transaminase 2** | **1** | **0** | **0** |
| **chr4_39447014_39447014** | **1,08E-10** | **6,32E-07** | **34,79695** | **Exon** | **8262** | **RPL9** | **ribosomalprotein L9** | **1** | **0** | **0** |
| **chr22_18039128_18039128** | **8,49E-09** | **2,75E-05** | **34,70085** | **DistalIntergenic** | **9743** | **LINC01634** | **long intergenic non-protein coding RNA 1634** | **0** | **0** | **1** |
| **chr9_122226146_122226146** | **1,58E-12** | **1,82E-08** | **34,58189** | **Promoter (<=1kb)** | **615** | **LHX6** | **LIM homeobox 6** | **1** | **0** | **0** |
| **chr18_13945478_13945478** | **2,88E-12** | **2,99E-08** | **34,58188** | **DistalIntergenic** | **-29771** | **MC2R** | **melanocortin 2 receptor** | **0** | **0** | **1** |
| **chr5_180313821_180313821** | **2,61E-12** | **2,80E-08** | **34,51128** | **Exon** | **10543** | **GFPT2** | **glutamine-fructose-6-phosphate transaminase 2** | **1** | **0** | **0** |
| **chr10_131969112_131969112** | **5,16E-08** | **0,000135** | **33,82609** | **3' UTR** | **12811** | **BNIP3** | **BCL2 interactingprotein 3** | **0** | **0** | **1** |
| **chr7_158102871_158102871** | **1,01E-12** | **1,26E-08** | **33,66004** | **Intron** | **102406** | **PTPRN2** | **protein tyrosine phosphatase receptor type N2** | **0** | **0** | **1** |
| **chr4_39447051_39447051** | **1,07E-09** | **4,48E-06** | **33,40586** | **Exon** | **8225** | **RPL9** | **ribosomalprotein L9** | **1** | **0** | **0** |
| **chr22_11600410_11600410** | **2,33E-13** | **4,03E-09** | **33,40322** | **DistalIntergenic** | **-639072** | **FRG1FP** | **FSHD region gene 1 family member F, pseudogene** | **0** | **1** | **0** |
| **chr20_7803868_7803868** | **2,94E-12** | **3,00E-08** | **33,33838** | **DistalIntergenic** | **136606** | **HAO1** | **hydroxyacidoxidase 1** | **0** | **0** | **1** |
| **chrX_324291_324291** | **4,18E-12** | **4,06E-08** | **33,00436** | **DistalIntergenic** | **-5472** | **GTPBP6** | **GTP bindingprotein 6 (putative)** | **1** | **0** | **0** |
| **chr9_122226131_122226131** | **1,12E-11** | **9,02E-08** | **32,88664** | **Promoter (<=1kb)** | **630** | **LHX6** | **LIM homeobox 6** | **1** | **0** | **0** |
| **chr3_127227058_127227058** | **3,00E-10** | **1,51E-06** | **32,61261** | **DistalIntergenic** | **33927** | **C3orf56** | **chromosome 3 open reading frame 56** | **0** | **0** | **1** |
| **chr10_131949138_131949138** | **1,69E-13** | **3,09E-09** | **32,61126** | **Intron** | **8531** | **PPP2R2D** | **protein phosphatase 2 regulatory subunit Bdelta** | **0** | **0** | **1** |
| **chr12_130483983_130483983** | **2,91E-07** | **0,000576** | **32,51058** | **Intron** | **-4967** | **RIMBP2** | **RIMS bindingprotein 2** | **0** | **0** | **1** |
| **chr9_122226154_122226154** | **2,79E-13** | **4,56E-09** | **32,48272** | **Promoter (<=1kb)** | **607** | **LHX6** | **LIM homeobox 6** | **1** | **0** | **0** |
| **chr11_97011586_97011586** | **5,52E-12** | **5,13E-08** | **32,33339** | **DistalIntergenic** | **503161** | **LOC105369443** | **uncharacterized LOC105369443** | **0** | **0** | **1** |
| **chr12_19814690_19814690** | **6,30E-09** | **2,13E-05** | **32,30638** | **Intron** | **-200090** | **LINC02398** | **long intergenic non-protein coding RNA 2398** | **0** | **0** | **1** |
| **chr20_7803849_7803849** | **1,36E-11** | **1,08E-07** | **32,22172** | **DistalIntergenic** | **136625** | **HAO1** | **hydroxyacidoxidase 1** | **0** | **0** | **1** |
| **chr20_29742670_29742670** | **1,65E-14** | **4,10E-10** | **32,05128** | **DistalIntergenic** | **-245491** | **FRG1EP** | **FSHD region gene 1 family member E, pseudogene** | **1** | **0** | **0** |
| **chr2_149320835_149320835** | **2,61E-07** | **0,00052** | **31,93146** | **Exon** | **-9150** | **LYPD6** | **LY6/PLAUR domain containing 6** | **0** | **1** | **0** |

*Legend:* Each row represents one dmCpGs which is identified by unique coordinates in the form of chr, start, end (hg38). The “p.value” is the unadjusted p value and “q.value” is the Benjamini-Hochberg adjusted p value; “meth.diff” column represents the magnitude of the difference in sequencing signal as a log2 fold change; “Genomic_context” specifies if the dmCpG falls within a portion of an annotated gene model. The distance to the nearest gene and the names of the nearest genes are provided along with the verlap with CGIs, shores, and other (shelves and open sea) The overlap is categorized by a pattern code: “1” represents the presence of the overlap and “0” represents the absence of overlap. This table illustrates the top 40 hyper-dmCpGs filtered by “meth.diff” (decreasing order). *Abbreviations:* CTRLs: Controls; CGIs: CpG Islands; dmCpGs: Differentially Methylated CpG Sites; PAH: Pulmonary Arterial Hypertension; TSS: Transcription Start Site.

**Supplementary Table 6**.Top 40 hypomethylated dmCpGs in PAH patients *vs.* CTRLs

| **Coordinates_dmCpGs** | **p.value** | **q.value** | **Meth.diff** | **Genomic**  **Context** | **Gene Distance**  **To TSS** | **Genes**  **Nearest** | **Gene Name** | **CGs** | **Shores** | **Other** |
| --- | --- | --- | --- | --- | --- | --- | --- | --- | --- | --- |
| **chr14_105077034_105077034** | **5,31E-05** | **0,036215** | **-20,0024** | **DistalIntergenic** | **-11589** | **GPR132** | **G protein-coupled receptor 132** | **0** | **0** | **1** |
| **chr19_1126802_1126802** | **1,91E-05** | **0,016828** | **-20,0535** | **Intron** | **5419** | **SBNO2** | **strawberrynotchhomolog 2** | **0** | **0** | **1** |
| **chr19_613349_613349** | **7,51E-06** | **0,007904** | **-20,0719** | **Exon** | **4648** | **POLRMT** | **RNA polymerasemitochondrial** | **1** | **0** | **0** |
| **chr3_6490870_6490870** | **5,81E-07** | **0,001021** | **-20,0915** | **Exon** | **154024** | **GRM7-AS3** | **GRM7 antisense RNA 3** | **0** | **0** | **1** |
| **chrX_142270426_142270426** | **1,85E-06** | **0,002584** | **-20,1164** | **DistalIntergenic** | **-65136** | **MAGEC2** | **MAGE family member C2** | **0** | **0** | **1** |
| **chr21_45348201_45348201** | **7,73E-05** | **0,048043** | **-20,1474** | **DistalIntergenic** | **-6211** | **LINC00316** | **long intergenic non-protein coding RNA 316** | **0** | **0** | **1** |
| **chr7_1555624_1555624** | **8,46E-07** | **0,001363** | **-20,1676** | **Promoter (<=1kb)** | **-93** | **TMEM184A** | **transmembrane protein 184A** | **0** | **0** | **1** |
| **chr6_126734626_126734626** | **5,73E-05** | **0,038387** | **-20,181** | **DistalIntergenic** | **249995** | **MIR588** | **microRNA 588** | **0** | **0** | **1** |
| **chr11_126698491_126698491** | **6,90E-05** | **0,044025** | **-20,201** | **Intron** | **42405** | **LOC101929427** | **uncharacterized LOC101929427** | **0** | **0** | **1** |
| **chr14_106288157_106288157** | **2,02E-06** | **0,002777** | **-20,2183** | **Promoter (<=1kb)** | **483** | **LINC00226** | **long intergenic non-protein coding RNA 226** | **0** | **1** | **0** |
| **chr13_114109893_114109893** | **2,40E-05** | **0,019958** | **-20,2521** | **Intron** | **22718** | **RASA3** | **RAS p21 proteinactivator 3** | **1** | **0** | **0** |
| **chr17_27197758_27197758** | **4,36E-05** | **0,031245** | **-20,2896** | **DistalIntergenic** | **96238** | **MIR4522** | **microRNA 4522** | **0** | **0** | **1** |
| **chr2_28545482_28545482** | **4,51E-07** | **0,000849** | **-20,4104** | **Intron** | **-3018** | **PLB1** | **phospholipase B1** | **0** | **0** | **1** |
| **chr10_27413846_27413846** | **5,70E-05** | **0,038236** | **-20,5602** | **Promoter (<=1kb)** | **522** | **PTCHD3** | **patched domain containing 3** | **1** | **0** | **0** |
| **chr7_135802119_135802119** | **2,00E-05** | **0,017349** | **-20,5929** | **DistalIntergenic** | **-53273** | **FAM180A** | **family with sequence similarity 180 member A** | **0** | **0** | **1** |
| **chr10_27413820_27413820** | **4,14E-05** | **0,030061** | **-20,6317** | **Promoter (<=1kb)** | **548** | **PTCHD3** | **patched domain containing 3** | **1** | **0** | **0** |
| **chr2_70797428_70797428** | **1,62E-08** | **4,77E-05** | **-20,6914** | **DistalIntergenic** | **-6785** | **FIGLA** | **folliculogenesis specific bHLH transcription factor** | **0** | **0** | **1** |
| **chr17_17229806_17229806** | **4,54E-05** | **0,032138** | **-20,7529** | **Promoter (<=1kb)** | **-329** | **FLCN** | **folliculin** | **0** | **0** | **1** |
| **chr11_70553610_70553610** | **4,77E-05** | **0,033296** | **-20,7577** | **Intron** | **-50751** | **SHANK2** | **SH3 and multiple ankyrin repeat domains 2** | **0** | **0** | **1** |
| **chr2_131724262_131724262** | **6,85E-05** | **0,04376** | **-20,7651** | **Promoter (1-2kb)** | **1845** | **C2orf27A** | **chromosome 2 open reading frame 27A** | **0** | **0** | **1** |
| **chr6_168126948_168126948** | **2,90E-05** | **0,023025** | **-20,7766** | **DistalIntergenic** | **-25437** | **FRMD1** | **FERM domain containing 1** | **0** | **1** | **0** |
| **chr7_155384382_155384382** | **2,76E-05** | **0,022338** | **-20,8144** | **Promoter (2-3kb)** | **2306** | **LOC100286906** | **uncharacterized LOC100286906** | **0** | **1** | **0** |
| **chr9_137519959_137519959** | **5,89E-07** | **0,001026** | **-20,8402** | **Exon** | **27415** | **PNPLA7** | **patatin like phospholipase domain containing 7** | **0** | **0** | **1** |
| **chr1_161198400_161198400** | **8,17E-06** | **0,008364** | **-20,8868** | **Promoter (<=1kb)** | **653** | **ADAMTS4** | **ADAM metallopeptidase with thrombospondin type 1 motif 4** | **0** | **0** | **1** |
| **chr9_134053541_134053541** | **7,48E-08** | **0,000187** | **-20,9316** | **Promoter (<=1kb)** | **599** | **BRD3** | **bromodomaincontaining 3** | **1** | **0** | **0** |
| **chr20_25300697_25300697** | **1,82E-05** | **0,016427** | **-20,9336** | **Promoter (<=1kb)** | **187** | **ABHD12** | **abhydrolase domain containing 12** | **0** | **0** | **1** |
| **chr7_5374386_5374386** | **4,40E-06** | **0,005143** | **-21,1365** | **Exon** | **-12359** | **TNRC18** | **trinucleotiderepeatcontaining 18** | **1** | **0** | **0** |
| **chr19_1832317_1832317** | **1,96E-06** | **0,002713** | **-21,2962** | **Intron** | **9117** | **LOC100288123** | **uncharacterized LOC100288123** | **0** | **0** | **1** |
| **chr1_117623070_117623070** | **1,71E-05** | **0,01558** | **-21,3014** | **Exon** | **17136** | **TENT5C** | **terminal nucleotidyltransferase 5C** | **0** | **0** | **1** |
| **chr9_133630195_133630195** | **7,73E-07** | **0,001274** | **-21,3077** | **Intron** | **-5153** | **DBH** | **dopamine beta-hydroxylase** | **0** | **0** | **1** |
| **chr11_1381808_1381808** | **1,60E-09** | **6,51E-06** | **-21,3169** | **DistalIntergenic** | **-8091** | **BRSK2** | **BR serine/threoninekinase 2** | **0** | **1** | **0** |
| **chr2_190710673_190710673** | **1,06E-06** | **0,001654** | **-21,3793** | **DistalIntergenic** | **50816** | **NAB1** | **NGFI-A bindingprotein 1** | **0** | **0** | **1** |
| **chr5_74766211_74766211** | **1,24E-06** | **0,001857** | **-21,4005** | **Promoter (<=1kb)** | **-780** | **NSA2** | **NSA2 ribosomebiogenesisfactor** | **0** | **1** | **0** |
| **chr19_1169022_1169022** | **6,06E-05** | **0,039916** | **-21,4503** | **Promoter (<=1kb)** | **124** | **SBNO2** | **strawberrynotchhomolog 2** | **1** | **0** | **0** |
| **chr20_58854188_58854188** | **4,49E-06** | **0,005219** | **-21,4521** | **Promoter (<=1kb)** | **19** | **GNAS** | **GNAS complex locus** | **1** | **0** | **0** |
| **chrX_155767171_155767171** | **7,71E-06** | **0,008056** | **-21,4695** | **Promoter (<=1kb)** | **-641** | **SPRY3** | **sprouty RTK signalingantagonist 3** | **0** | **0** | **1** |
| **chr16_48852571_48852571** | **3,50E-06** | **0,004326** | **-21,5053** | **DistalIntergenic** | **-232423** | **N4BP1** | **NEDD4 bindingprotein 1** | **0** | **0** | **1** |
| **chr17_881397_881397** | **2,94E-06** | **0,003724** | **-21,5563** | **Intron** | **15617** | **NXN** | **nucleoredoxin** | **0** | **0** | **1** |
| **chr2_127641558_127641558** | **1,03E-05** | **0,010197** | **-21,6813** | **Promoter (<=1kb)** | **573** | **LIMS2** | **LIM zinc finger domain containing 2** | **0** | **0** | **1** |
| **chr5_172434459_172434459** | **7,08E-05** | **0,044859** | **-21,7228** | **Intron** | **19849** | **SH3PXD2B** | **SH3 and PX domains 2B** | **0** | **0** | **1** |

*Legend:* Each row represents one dmCpGs which is identified by unique coordinates in the form of chr, start, end (hg38). The “pvalue” is the unadjusted p value and “q.value” is the Benjamini-Hochberg adjusted p value; “meth.diff” column represents the magnitude of the difference in sequencing signal as a log2 fold change; “Genomic_context” specifies if the dmCpG falls within a portion of an annotated gene model. The distance to the nearest gene and the names of the nearest genes are provided along with the overlap with CGIs, shores, and other (shelves and open sea) The overlap is categorized by a pattern code: “1” represents the presence of the overlap and “0” represents the absence of overlap.This table illustrates the top 40 hypo-dmCpGs filtered by “meth.diff” (decreasing order).*Abbreviations:* CTRLs: Controls; CGIs: CpG Islands; dmCpGs: Differentially Methylated CpG Sites; PAH: Pulmonary Arterial Hypertension; TSS: Transcription Start Site.

**Supplementary Table 7**. Hub nodes of the hypermethylated lung-specific DNA methylation-PPI network

| **Id** | **Gene Name (Nodes)** | **Genomic Localization** | **Degree** | **Betweenness** |
| --- | --- | --- | --- | --- |
| 4163 | **MCC** | Intron | 128 | 142741.27 |
| 5105 | **PCK1** | Downstream | 73 | 82913.79 |
| 1789 | **DNMT3B** | Promoter | 53 | 54435.92 |
| 6133 | **RPL9** | Exon | 49 | 63609.75 |
| 9612 | **NCOR2** | Promoter | 47 | 47768.03 |
| 57521 | **RPTOR** | Promoter | 46 | 49366.41 |
| 1488 | **CTBP2** | Promoter | 43 | 41932.69 |
| 2516 | **NR5A1** | Intron | 34 | 28064.49 |
| 547 | **KIF1A** | Intron | 25 | 23566.92 |
| 56829 | **ZC3HAV1** | Exon | 21 | 36859.59 |
| 5317 | **PKP1** | Promoter | 19 | 13059.64 |
| 23677 | **SH3BP4** | Promoter | 16 | 13513.91 |
| 9935 | **MAFB** | Distal | 16 | 12919.82 |
| 339390 | **CLEC4G** | Promoter | 16 | 12764.2 |
| 7050 | **TGIF1** | Intron | 15 | 32169.72 |
| 64759 | **TNS3** | Exon | 15 | 14007.63 |
| 10865 | **ARID5A** | Distal | 15 | 8828.22 |
| 4929 | **NR4A2** | Promoter | 15 | 7667.81 |
| 351 | **APP** | … | 14 | 82195.43 |
| 55743 | **CHFR** | Intron | 14 | 13278.5 |
| 2321 | **FLT1** | Intron | 14 | 12867 |
| 55844 | **PPP2R2D** | Intron | 13 | 9623.96 |
| 5230 | **PGK1** | Promoter | 13 | 8879.84 |
| 4145 | **MATK** | Exon | 13 | 8630.43 |
| 8664 | **EIF3D** | Distal | 12 | 16528.79 |
| 8624 | **PSMG1** | Distal | 11 | 17767.06 |
| 23332 | **CLASP1** | Distal | 11 | 8901.88 |
| 2195 | **FAT1** | Intron | 10 | 8934.58 |

*Legend***:** We show the hub nodes into the lung-specific DNA methylation-PPI subnetwork obtained with the list of hypermethylated DMGs. For each node, we indicate the unique ID, Gene Name, Degree (the total number of links with other nodes in the subnetwork), and Betweenness Centrality (the number of times a node acts as a bridge along the shortest path between two other nodes).*Abbreviations:* PPI: Protein-Protein Interactions.

**Supplementary Table 8.** Hub nodes of the hypomethylated lung-specific DNA methylation-PPI network

| **Id** | **Gene Name** | **Genomic Localization** | **Degree** | **Betweenness** |
| --- | --- | --- | --- | --- |
| 5925 | **RB1** | Exon | 95 | 92862.92 |
| 1894 | **ECT2** | Distal | 91 | 67434.45 |
| 9021 | **SOCS3** | Promoter | 77 | 78238.92 |
| 821 | **CANX** | Distal | 50 | 39030.38 |
| 274 | **BIN1** | Distal | 36 | 47201.45 |
| 3480 | **IGF1R** | Intron | 35 | 44865.16 |
| 4172 | **MCM3** | Distal | 29 | 32507.71 |
| 57504 | **MTA3** | Intron | 26 | 28264.37 |
| 9046 | **DOK2** | Distal | 23 | 16996.37 |
| 6857 | **SYT1** | Exon | 23 | 15580.59 |
| 2778 | **GNAS** | Promoter | 21 | 15710.35 |
| 2317 | **FLNB** | Intron | 20 | 15090.2 |
| 6154 | **RPL26** | Exon | 20 | 11446.04 |
| 55211 | **DPPA4** | Promoter | 17 | 8443.68 |
| 3925 | **STMN1** | Promoter | 16 | 7574.61 |
| 3911 | **LAMA5** | Promoter | 15 | 10196.42 |
| 10291 | **SF3A1** | Promoter | 14 | 10762.79 |
| 115992 | **RNF166** | Promoter | 14 | 6818 |
| 682 | **BSG** | Promoter | 13 | 9258.45 |
| 1734 | **DIO2** | Intron | 13 | 7344.78 |
| 54941 | **RNF125** | Distal | 12 | 15206.29 |
| 10395 | **DLC1** | Promoter | 12 | 8591.95 |
| 4205 | **MEF2A** | Distal | 12 | 8153.86 |
| 51438 | **MAGEC2** | Distal | 11 | 7565 |
| 54039 | **PCBP3** | Intron | 11 | 7565 |

*Legend*: We show the hub nodes into the lung-specific DNA methylation-PPI subnetwork obtained with the list of hypomethylated DMGs. For each node, we indicate the unique ID, Gene Name, Degree (the total number of links with other nodes in the subnetwork), and Betweenness Centrality (the number of times a node acts as a bridge along the shortest path between two other nodes). *Abbreviations:* PPI: Protein-Protein Interactions.

**Supplementary Table 9.** The PAH subnetwork

| **DMGs** | **dmCpG_ID** | **meth.diff** | **Methylation**  **status** | **Genomic Context** | **CpGi** | **shores** | **other** |
| --- | --- | --- | --- | --- | --- | --- | --- |
| **Hubs** | | | | | | | |
| ***SOCS3*** | chr17_78358867_78358867 | -24,6154 | hypo | Promoter (1-2kb) | 1 | 0 | 0 |
| ***SOCS3*** | chr17_78358875_78358875 | -22,3932 | hypo | Promoter (1-2kb) | 1 | 0 | 0 |
| ***GNAS*** | chr20_58854188_58854188 | -21,4521 | hypo | Promoter (<=1kb) | 1 | 0 | 0 |
| ***ITGAL*** | chr16_30474222_30474222 | -21,9728 | hypo | Promoter (<=1kb) | 1 | 0 | 0 |
| ***ITGAL*** | chr16_30474235_30474235 | -22,7246 | hypo | Promoter (<=1kb) | 1 | 0 | 0 |
| ***ITGAL*** | chr16_30474222_30474222 | -21,9728 | hypo | Promoter (<=1kb) | 1 | 0 | 0 |
| ***ITGAL*** | chr16_30474235_30474235 | -22,7246 | hypo | Promoter (<=1kb) | 1 | 0 | 0 |
| ***NFIC*** | chr19_3367789_3367789 | 20,10555 | hyper | Promoter (1-2kb) | 0 | 1 | 0 |
| ***NCOR2*** | chr12_124354604_124354604 | 24,0238 | hyper | Promoter (2-3kb) | 0 | 0 | 1 |
| **Non Hubs** | | | | | | | |
| ***NR4A2*** | chr2_156329514_156329514 | 24,38819 | hyper | Promoter (<=1kb) | 1 | 0 | 0 |
| ***NR4A2*** | chr2_156329533_156329533 | 20,60319 | hyper | Promoter (<=1kb) | 1 | 0 | 0 |
| ***NR4A2*** | chr2_156329545_156329545 | 24,06281 | hyper | Promoter (<=1kb) | 1 | 0 | 0 |
| ***NR4A2*** | chr2_156329842_156329842 | 30,56818 | hyper | Promoter (<=1kb) | 1 | 0 | 0 |
| ***GRM2*** | chr3_51707336_51707336 | 21,55418 | hyper | Promoter (<=1kb) | 1 | 0 | 0 |
| ***PGK1*** | chrX_78104084_78104084 | 20,73477 | hyper | Promoter (<=1kb) | 1 | 0 | 0 |
| ***PGK1*** | chrX_78104127_78104127 | 20,51496 | hyper | Promoter (<=1kb) | 1 | 0 | 0 |
| ***STMN1*** | chr1_25909325_25909325 | -26,0526 | hypo | Promoter (2-3kb) | 0 | 0 | 1 |
| ***LIMS2*** | chr2_127641558_127641558 | -21,6813 | hypo | Promoter (<=1kb) | 0 | 0 | 1 |

*Legend*: Each row indicates the unique chromosomal position, the met.diff, the methylation status, the genomic localization, and the distribution in the CGI context of each dmCpGs. *Abbreviations:* dmCpGs: Differentially Methylated CpG Sites; *ITGAL*: Integrin Subunit Alpha L; CGI: CpG Island; *GNAS*: Guanine Nucleotide Binding Protein (G Protein), Alpha Stimulating Activity; *GRM2*: Glutamate Metabotropic Receptor 2; *LIMS2*: LIM Zinc Finger Domain Containing 2; *NCOR2*: Nuclear Receptor Corepressor 2; *NFIC*: Nuclear Factor I C; *NR4A2*: Nuclear Receptor Subfamily 4 Group A Member 2; PAH: Pulmonary Arterial Hypertension; *PGK1*: Phosphoglycerate Kinase 1; *SOCS3*: Suppressor of Cytokine Signaling 3; *STMN1*: Stathmin.

**Supplementary Table 10**. Enrichment pathway analysisof the PAH Subnetwork

| **Pathway** | **Genes** | **P** | **FDR** |
| --- | --- | --- | --- |
| **Growthhormone receptor signaling** | JAK2,MAPK1,MAPK3,STAT3,STAT5A,STAT5B,SOCS3 | 1.91e-10 | 2.6e-07 |
| **Interleukin-6 signaling** | JAK2,STAT3,SOCS3,IL6,IL6R,PTPN11 | 3.71e-10 | 2.6e-07 |
| **Signaling by Interleukins** | JAK2,MAPK1,MAPK3,STAT3,STAT5A,STAT5B,SOCS3,IL6,IL6R,PTPN11, PTK2B | 5.7e-10 | 2.66e-07 |
| **Signaling by SCF-KIT** | JAK2,MAPK1,MAPK3,STAT3,STAT5A,STAT5B,PTPN11,AKT1,MTOR,NR4A1,SRC | 5.01e-09 | 1.76e-06 |
| **Cytokine Signaling in Immune system** | JAK2,MAPK1,MAPK3,STAT3,STAT5A,STAT5B,SOCS3,IL6,IL6R,PTPN11,PTK2B,ICAM1,IFNA1,IFNB1 | 1.19e-08 | 3.34e-06 |
| **Signaling by ERBB4** | JAK2,MAPK1,MAPK3,STAT5A,STAT5B,PTPN11,AKT1,MTOR,NR4A1,ESR1 | 1.31e-07 | 3.06e-05 |
| **SignalTransduction** | JAK2,MAPK1,MAPK3,STAT3,STAT5A,STAT5B,PTPN11,AKT1,MTOR,NR4A1,SRC,ICAM1,ESR1,AGTR1,CCR5,EDN1,EDNRA,GNAS,GRM2,ITGAL,SMAD3,SMAD4,NOTCH1,NOTCH3,RELA,VIP,VWF,CXCR4,NCOR2,GPBAR1 | 1.9e-07 | 3.81e-05 |
| **Downstream signaltransduction** | MAPK1,MAPK3,STAT3,STAT5A,STAT5B,PTPN11,AKT1,MTOR,NR4A1,SRC | 2.53e-07 | 4.43e-05 |
| **Hemostasis** | JAK2,MAPK1,MAPK3,PTPN11,AKT1,SRC,IFNA1,IFNB1,GNAS,ITGAL,VWF,ACTB,CAV1,FLNA,SOD1,TP53 | 4.68e-07 | 7.29e-05 |
| **Interleukin-2 signaling** | JAK2,MAPK1,MAPK3,STAT5A,STAT5B,PTK2B | 5.53e-07 | 7.32e-05 |

*Legend*: For each significant pathway, we indicate the official name, the list of genes, pvalue, and the adjusted p.value (FDR).

**Supplementary Table 11**.Characteristics of validation set (EPIC)

| **Variable** | **PAH (N=5)** | **CTRLs (N=4)** |
| --- | --- | --- |
| **Age, y** | 71.0± 4.95 | 28.25± 4.61 |
| **Female** | 3 (60.0) | 2 (50.0) |
| **PAH phenotype** |  |  |
| **IPAH** | 3 (60.0) |  |
| **PAH-SSc** | 1 (20.0) |  |
| **PAH-CHD** | 1 (20.0) |  |
| **NYHA class** |  |  |
| **II** | 2 (40.0) |  |
| **III** | 3 (60.0) |  |
| **Time of biospecimen collection** |  |  |
| **First diagnosis** | 2 (40.0) |  |
| **Follow-up**  (within 1 year from diagnosis) | 3 (60.0) |  |
| **Invasive hemodynamics** |  |  |
| **RAP, mmHg** | 4.60± 3.71 |  |
| **mPAP, mmHg** | 39.0± 13.5 |  |
| **PCWP, mmHg** | 9.80± 0.83 |  |
| **PVR** | 8.78 ± 5.82 |  |
| **Cardiac index, L/min/m2** | 2. 04± 0.35 |  |
| **Risk category** |  |  |
| **Intermediate-risk** | 2 (40.0) |  |
| **High-risk** | 3 (60.0) |  |

Data are n (%) or mean ± standard deviation (SD). *Abbreviations:* CI: Cardiac Index; CTRL: Healthy Control; IPAH: Idiopathic PAH; mPAP: Mean Pulmonary Arterial Pressure; NYHA: New York Heart Association; PAH-CHD: PAH Associated with Congenital Heart Disease; PAH-SSc: PAH Associated with Systemic Sclerosis: PCWP: Pulmonary Capillary Wedge Pressure; PVR: Pulmonary Vascular Resistance; RAP: Right Atrial Pressure; RRBS: Reduced Representation Bisulfite Sequencing

**Supplementary Table 12**.Coordinates of common dmCpGs between Array 1 and Array 2

| **CG_ID** | **IlmnID_Array** | **DiffMethArray2** | **Methylation statusArray2** | **DiffMethArray1** | **Methylation status Array1** | **Genomic**  **Localization** |
| --- | --- | --- | --- | --- | --- | --- |
| ***SOCS3*** | | | | | | |
| **chr17_78358540** | cg18181703 | -0,007057847 | hypo | -0,093328497 | hypo | Body |
| **chr17_78358853** | cg11047325 | -0,019540591 | hypo | -0,149882186 | hypo | Body |
| **chr17_78358867** | dmCpG_RRBS |  |  |  |  |  |
| **chr17_78358875** | dmCpG_RRBS |  |  |  |  |  |
| **chr17_78359065** | cg10508317 | -0,023009188 | hypo | -0,004328531 | hypo | Body |
| **chr17_78359207** | cg10279487 | -0,005323752 | hypo | -0,001385779 | hypo | 5'UTR |
| **chr17_78360071** | cg21985352 | -0,014828996 | hypo | -0,007258503 | hypo | 1stExon;5'UTR |
| **chr17_78360151** | cg01897823 | -0,000202433 | hypo | -0,010198397 | hypo | TSS200 |
| **chr17_78360733** | cg21500342 | -0,003493831 | hypo | -0,005717779 | hypo | TSS1500 |
| **chr17_78360919** | cg09642498 | -0,010040781 | hypo | -0,003316137 | hypo | TSS1500 |
| **chr17_78361151** | cg03752138 | -0,090519421 | hypo | -0,009072941 | hypo | TSS1500 |
| ***GNAS*** | | | | | | |
| **chr20_58839004** | cg25090051 | -0,013062165 | hypo | -0,000111815 | hypo | TSS1500 |
| **chr20_58839541** | cg21330323 | -0,018338055 | hypo | -0,015831416 | hypo | TSS200 |
| **chr20_58839690** | cg17986444 | -0,025806464 | hypo | -0,026191238 | hypo | TSS200 |
| **chr20_58839829** | cg06163629 | -0,020993712 | hypo | -0,011263931 | hypo | 1stExon;5'UTR |
| **chr20_58840900** | cg15725616 | -0,025383371 | hypo | -0,00911633 | hypo | TSS1500;3'UTR |
| **chr20_58840923** | cg18035618 | -0,000632338 | hypo | -0,007043696 | hypo | TSS1500;3'UTR |
| **chr20_58849466** | cg10546626 | -0,01820004 | hypo | -0,013315712 | hypo | 3'UTR |
| **chr20_58851876** | cg21938532 | -0,000234338 | hypo | -0,006783879 | hypo | TSS1500;3'UTR |
| **chr20_58851880** | cg03606258 | -0,035324834 | hypo | -0,032367945 | hypo | TSS1500;3'UTR |
| **chr20_58851962** | cg05309239 | -0,001560123 | hypo | -0,015740645 | hypo | TSS1500;3'UTR |
| **chr20_58852918** | cg19640589 | -0,009941963 | hypo | -0,051377622 | hypo | TSS200;3'UTR |
| **chr20_58853254** | cg21809160 | -0,014535412 | hypo | -0,003243505 | hypo | 5'UTR;1stExon;3'UTR |
| **chr20_58853260** | cg09583957 | -0,03306313 | hypo | -0,03358707 | hypo | 5'UTR;1stExon;3'UTR |
| **chr20_58855078** | cg26711395 | -0,017218792 | hypo | -0,012613877 | hypo | 1stExon;3'UTR;5'UTR |
| **chr20_58855608** | cg14203179 | -0,00033617 | hypo | -0,031624147 | hypo | 3'UTR;Body |
| **chr20_58856147** | cg09437522 | -0,022947004 | hypo | -0,008689116 | hypo | 3'UTR;Body |
| **chr20_58860091** | cg15122327 | -0,019760858 | hypo | -0,011189345 | hypo | 3'UTR;Body |
| **chr20_58888517** | cg17334845 | -0,009419273 | hypo | -0,005263486 | hypo | 3'UTR;TSS1500;Body |
| **chr20_58888560** | cg26767990 | -0,035189069 | hypo | -0,028785855 | hypo | 3'UTR;TSS1500;Body |
| **chr20_58888598** | cg17652507 | -0,004953641 | hypo | -0,027743944 | hypo | 3'UTR;TSS1500;Body |
| **chr20_58888603** | cg22407822 | -0,012898829 | hypo | -0,039630819 | hypo | 3'UTR;TSS1500;Body |
| **chr20_58888708** | cg25308079 | -0,020570426 | hypo | -0,026514791 | hypo | 3'UTR;TSS1500;Body |
| **chr20_58888728** | cg15222215 | -0,011466505 | hypo | -0,023836951 | hypo | 3'UTR;TSS1500;Body |
| **chr20_58888851** | cg05926269 | -0,028814259 | hypo | -0,011742863 | hypo | 3'UTR;TSS1500;Body |
| **chr20_58888870** | cg03821543 | -0,000847084 | hypo | -0,044582048 | hypo | 3'UTR;TSS1500;Body |
| **chr20_58888919** | cg01538522 | -0,002946859 | hypo | -0,01624291 | hypo | 3'UTR;TSS1500;Body |
| **chr20_58888929** | cg00267746 | -0,024935251 | hypo | -0,027193951 | hypo | TSS200;3'UTR;Body |
| **chr20_58888947** | cg23159236 | -0,034524226 | hypo | -0,040007073 | hypo | TSS200;3'UTR;Body |
| **chr20_58889074** | cg22798925 | -0,024094811 | hypo | -0,032761424 | hypo | TSS200;3'UTR;Body |
| **chr20_58890084** | cg20018057 | -0,014856701 | hypo | -0,009361665 | hypo | 3'UTR;TSS1500;Body |
| **chr20_58890640** | cg18300848 | -0,004002503 | hypo | -0,006562961 | hypo | 3'UTR;TSS1500;Body |
| **chr20_58890718** | cg07516978 | -0,010488024 | hypo | -0,012830066 | hypo | 3'UTR;TSS1500;Body |
| **chr20_58890720** | cg13231951 | -0,014772935 | hypo | -0,003274181 | hypo | 3'UTR;TSS1500;Body |
| **chr20_58891082** | cg18668503 | -0,01950611 | hypo | -0,013705955 | hypo | 3'UTR;TSS1500;Body |
| **chr20_58891850** | cg06147822 | -0,005075079 | hypo | -0,000449441 | hypo | 3'UTR;1stExon;Body |
| **chr20_58896605** | cg11021321 | -0,017577259 | hypo | -0,012088099 | hypo | Body;3'UTR |
| **chr20_58910775** | cg03673016 | -0,001692085 | hypo | -0,026182926 | hypo | Body;3'UTR |
| **chr20_58910782** | cg13353325 | -9,10E-05 | hypo | -0,00403394 | hypo | Body;3'UTR |
| **chr20_58910885** | cg20083839 | -0,011440811 | hypo | -0,073659936 | hypo | 3'UTR;Body |
| ***ITGAL*** | | | | | | |
| **chr16_30471910** | cg02606575 | -0,018610699 | hypo | -0,013454048 | hypo | TSS1500 |
| **chr16_30472675** | cg10522125 | -0,000726277 | hypo | -0,009947326 | hypo | 5'UTR;1stExon |
| **chr16_30472897** | cg14176836 | -0,009145931 | hypo | -0,008914312 | hypo | 1stExon |
| **chr16_30479347** | cg25844028 | -0,019508901 | hypo | -0,019592856 | hypo | ExonBnd;Body |
| **chr16_30481502** | cg13337899 | -0,098965159 | hypo | -0,014778003 | hypo | Body |
| **chr16_30481584** | cg02976720 | -0,055501461 | hypo | -0,049954532 | hypo | ExonBnd;Body |
| **chr16_30483226** | cg00402343 | -0,003620002 | hypo | -0,00494072 | hypo | Body |
| **chr16_30483730** | cg09798624 | -0,004019061 | hypo | -0,013480992 | hypo | Body |
| **chr16_30485925** | cg23954865 | -0,14110129 | hypo | -0,008193251 | hypo | Body |
| **chr16_30490458** | cg10872919 | -0,095511804 | hypo | -0,0046324 | hypo | Body |
| **chr16_30496299** | cg26898455 | -0,002283105 | hypo | -0,010514066 | hypo | ExonBnd;Body |
| **chr16_30499817** | cg10236271 | -0,019575963 | hypo | -0,030571885 | hypo | Body |
| **chr16_30509248** | cg14794592 | -0,05902469 | hypo | -0,049341914 | hypo | Body |
| **chr16_30517657** | cg19600209 | -0,015202844 | hypo | -0,013740503 | hypo | ExonBnd;Body |
| **chr16_30519711** | cg14021764 | -0,007264435 | hypo | -0,016127852 | hypo | Body |
| ***NCOR2*** | | | | | | |
| **chr12_124324477** | cg27103591 | 0,009543515 | hyper | 0,00331439 | hyper | 3'UTR |
| **chr12_124324551** | cg14219896 | 0,010803111 | hyper | 0,017773605 | hyper | 3'UTR |
| **chr12_124324618** | cg14570145 | 0,00023427 | hyper | 0,078237558 | hyper | 3'UTR |
| **chr12_124325253** | cg22466209 | 0,001038705 | hyper | 0,001799806 | hyper | 3'UTR |
| **chr12_124325627** | cg13127920 | 0,064445576 | hyper | 0,548915993 | hyper | Body |
| **chr12_124326362** | cg07968091 | 0,00264251 | hyper | 0,052990945 | hyper | Body |
| **chr12_124328755** | cg20251110 | 0,067619766 | hyper | 0,015161553 | hyper | Body |
| **chr12_124328775** | cg00254608 | 0,065107886 | hyper | 0,032051617 | hyper | Body |
| **chr12_124332507** | cg27641233 | 0,012723627 | hyper | 0,03742184 | hyper | Body |
| **chr12_124336642** | cg19215678 | 0,003336297 | hyper | 0,002013464 | hyper | Body |
| **chr12_124337448** | cg09694328 | 0,000552826 | hyper | 0,025976058 | hyper | Body |
| **chr12_124337459** | cg17120358 | 0,14752942 | hyper | 0,004042853 | hyper | Body |
| **chr12_124338088** | cg20337110 | 0,005855974 | hyper | 0,003374487 | hyper | Body |
| **chr12_124338713** | cg22691028 | 0,005081904 | hyper | 0,011782761 | hyper | Body |
| **chr12_124342245** | cg22377629 | 0,002864965 | hyper | 0,001183799 | hyper | Body |
| **chr12_124342769** | cg23878260 | 0,153499789 | hyper | 0,015642047 | hyper | Body |
| **chr12_124343153** | cg06024623 | 0,004908516 | hyper | 0,001805689 | hyper | Body |
| **chr12_124343183** | cg02359414 | 0,007668759 | hyper | 0,020066083 | hyper | Body |
| **chr12_124344897** | cg21131796 | 0,008676101 | hyper | 0,00237998 | hyper | Body |
| **chr12_124346883** | cg02341605 | 0,023535744 | hyper | 0,02171382 | hyper | Body |
| **chr12_124348209** | cg27653901 | 0,020170011 | hyper | 0,000478357 | hyper | Body |
| **chr12_124349389** | cg20121920 | 0,004924255 | hyper | 8,21E-05 | hyper | Body |
| **chr12_124354604** | dmCpG_RRBS |  |  |  |  |  |
| **chr12_124360215** | cg06130683 | 0,014621836 | hyper | 0,005949454 | hyper | Body |
| **chr12_124369736** | cg16496462 | 0,014330487 | hyper | 0,01877606 | hyper | Body |
| **chr12_124371830** | cg10082088 | 0,004864626 | hyper | 0,022262329 | hyper | Body |
| **chr12_124373392** | cg27615368 | 0,006389082 | hyper | 0,002401059 | hyper | Body |
| **chr12_124377317** | cg11231155 | 0,176790795 | hyper | 0,365789017 | hyper | Body |
| **chr12_124378532** | cg12255505 | 0,029578742 | hyper | 0,033111041 | hyper | Body |
| **chr12_124382957** | cg04884232 | 0,003871774 | hyper | 0,000296559 | hyper | Body |
| **chr12_124388801** | cg24975642 | 0,052056607 | hyper | 0,081480358 | hyper | Body |
| **chr12_124388969** | cg13031847 | 0,040407431 | hyper | 0,15718393 | hyper | Body |
| **chr12_124389127** | cg12154538 | 0,061598533 | hyper | 0,117436975 | hyper | Body |
| **chr12_124389132** | cg04052996 | 0,063992655 | hyper | 0,087323683 | hyper | Body |
| **chr12_124389461** | cg14951136 | 0,028681801 | hyper | 0,058037995 | hyper | Body |
| **chr12_124391524** | cg12349623 | 0,016131458 | hyper | 0,110422268 | hyper | Body |
| **chr12_124395714** | cg25754673 | 0,002022531 | hyper | 0,006254839 | hyper | Body |
| **chr12_124409010** | cg06881647 | 0,001935077 | hyper | 0,019451908 | hyper | Body |
| **chr12_124409103** | cg11437331 | 0,017143077 | hyper | 0,01683969 | hyper | Body |
| **chr12_124411533** | cg23880533 | 0,009719555 | hyper | 0,030878403 | hyper | Body |
| **chr12_124412068** | cg14031846 | 0,006560013 | hyper | 0,013888212 | hyper | Body |
| **chr12_124413323** | cg11210443 | 0,003061732 | hyper | 0,011198731 | hyper | Body |
| **chr12_124416878** | cg02758507 | 0,008185062 | hyper | 0,013332531 | hyper | Body |
| **chr12_124420077** | cg13697207 | 0,026373299 | hyper | 0,067534968 | hyper | Body |
| **chr12_124420155** | cg24446009 | 0,011791945 | hyper | 0,011126285 | hyper | Body |
| **chr12_124420386** | cg22265294 | 0,007896145 | hyper | 0,04728135 | hyper | Body |
| **chr12_124420684** | cg17429587 | 0,029683273 | hyper | 0,000880155 | hyper | Body |
| **chr12_124423566** | cg08976554 | 0,003899749 | hyper | 0,010378706 | hyper | Body |
| **chr12_124426733** | cg27547841 | 0,002749874 | hyper | 0,012689411 | hyper | Body |
| **chr12_124426812** | cg01485790 | 0,003547847 | hyper | 0,003419439 | hyper | Body |
| **chr12_124426898** | cg12509268 | 0,013305701 | hyper | 0,015405022 | hyper | Body |
| **chr12_124427636** | cg17154218 | 0,013854475 | hyper | 0,005941928 | hyper | Body |
| **chr12_124430639** | cg14185801 | 0,001470321 | hyper | 0,00866142 | hyper | Body |
| **chr12_124442048** | cg14466753 | 0,083555163 | hyper | 0,052233822 | hyper | Body |
| **chr12_124443006** | cg19350162 | 0,000944167 | hyper | 0,022800689 | hyper | Body |
| **chr12_124445044** | cg05596926 | 0,019460091 | hyper | 0,043870236 | hyper | Body |
| **chr12_124445158** | cg08143046 | 0,00213302 | hyper | 0,003042994 | hyper | Body |
| **chr12_124445445** | cg03048372 | 0,009264627 | hyper | 0,022144034 | hyper | Body |
| **chr12_124445614** | cg16878595 | 0,013784297 | hyper | 0,000501222 | hyper | Body |
| **chr12_124448842** | cg06196466 | 0,001105221 | hyper | 0,019893594 | hyper | Body |
| **chr12_124463522** | cg03406367 | 0,037142201 | hyper | 0,000991982 | hyper | Body |
| **chr12_124469378** | cg09267427 | 0,059351049 | hyper | 0,048430119 | hyper | Body |
| **chr12_124477881** | cg23180506 | 0,019914241 | hyper | 0,058360304 | hyper | Body |
| **chr12_124483850** | cg09085501 | 0,001273561 | hyper | 0,007831976 | hyper | Body |
| **chr12_124486565** | cg13982220 | 0,001388338 | hyper | 0,01437986 | hyper | Body |
| **chr12_124494025** | cg27067412 | 0,097578706 | hyper | 0,001775772 | hyper | Body |
| **chr12_124497456** | cg08192337 | 0,032555726 | hyper | 0,006439295 | hyper | 5'UTR |
| **chr12_124500726** | cg14256268 | 0,006819002 | hyper | 0,005246682 | hyper | 5'UTR |
| **chr12_124501109** | cg15627464 | 0,049618449 | hyper | 0,019465155 | hyper | 5'UTR |
| **chr12_124504732** | cg07928378 | 0,035798304 | hyper | 0,044085105 | hyper | 5'UTR |
| **chr12_124506396** | cg22700848 | 0,011403454 | hyper | 0,098327822 | hyper | 5'UTR |
| **chr12_124506541** | cg04403415 | 0,009240708 | hyper | 0,132710882 | hyper | 5'UTR |
| **chr12_124511517** | cg18906815 | 0,008729404 | hyper | 0,019354037 | hyper | 5'UTR |
| **chr12_124516188** | cg12828588 | 0,004422624 | hyper | 0,012502479 | hyper | 5'UTR |
| **chr12_124516221** | cg26641022 | 0,008069661 | hyper | 0,010079915 | hyper | 5'UTR |
| **chr12_124517645** | cg22828383 | 0,020206736 | hyper | 0,066134859 | hyper | 5'UTR |
| **chr12_124524527** | cg08965565 | 0,006027802 | hyper | 0,207007372 | hyper | 5'UTR |
| **chr12_124524596** | cg07747189 | 0,002456722 | hyper | 0,016735821 | hyper | 5'UTR |
| **chr12_124527859** | cg06955420 | 0,009568555 | hyper | 0,030504463 | hyper | 5'UTR |
| **chr12_124528632** | cg15837490 | 0,003594023 | hyper | 0,003599311 | hyper | 5'UTR |
| **chr12_124535501** | cg23611643 | 0,006681429 | hyper | 0,0174132 | hyper | 5'UTR |
| **chr12_124535851** | cg20086252 | 0,001420358 | hyper | 0,007919751 | hyper | 5'UTR |
| **chr12_124535914** | cg13857025 | 0,014788457 | hyper | 0,01872743 | hyper | TSS1500 |
| **chr12_124536900** | cg23237987 | 0,047019684 | hyper | 0,008831854 | hyper | 5'UTR |
| **chr12_124537561** | cg00872086 | 0,075875869 | hyper | 0,032759513 | hyper | 5'UTR |
| **chr12_124554023** | cg04315470 | 0,01618434 | hyper | 0,039762682 | hyper | 5'UTR |
| **chr12_124562281** | cg18278347 | 0,00236051 | hyper | 0,027715268 | hyper | 5'UTR |
| ***NFIC*** | | | | | | |
| **chr19_3358184** | cg03583050 | 0,015109827 | hyper | 0,060790137 | hyper | TSS1500 |
| **chr19_3358994** | cg26044825 | 0,004776289 | hyper | 0,005066646 | hyper | TSS1500 |
| **chr19_3359039** | cg13726332 | 0,004759204 | hyper | 0,008134047 | hyper | TSS1500 |
| **chr19_3360282** | cg00416384 | 0,010725911 | hyper | 0,000618789 | hyper | Body;Body |
| **chr19_3366228** | cg01325908 | 0,004102082 | hyper | 0,054105407 | hyper | TSS1500;Body |
| **chr19_3366629** | cg10540861 | 0,002243141 | hyper | 0,005053307 | hyper | 5'UTR;1stExon;Body |
| **chr19_3367380** | cg10712606 | 0,000700076 | hyper | 0,154457838 | hyper | Body |
| **chr19_3369704** | cg03381209 | 0,00538629 | hyper | 0,044813157 | hyper | Body |
| **chr19_3369716** | cg10688516 | 0,000995409 | hyper | 0,056617162 | hyper | Body |
| **chr19_3373821** | cg09925682 | 0,042965315 | hyper | 0,017739637 | hyper | Body |
| **chr19_3382129** | cg22731204 | 0,002986357 | hyper | 0,002151617 | hyper | Body |
| **chr19_3384856** | cg14543226 | 0,044121267 | hyper | 0,084258737 | hyper | Body |
| **chr19_3385007** | cg08927067 | 0,058708633 | hyper | 0,040500131 | hyper | Body |
| **chr19_3388323** | cg00518009 | 0,014335799 | hyper | 0,011156171 | hyper | Body |
| **chr19_3395653** | cg07818063 | 0,006530446 | hyper | 0,042973496 | hyper | Body |
| **chr19_3396625** | cg09159514 | 0,002454075 | hyper | 0,011672177 | hyper | Body |
| **chr19_3407725** | cg10051614 | 0,023136634 | hyper | 0,03453833 | hyper | Body |
| **chr19_3434932** | cg26026416 | 0,003428914 | hyper | 0,005194763 | hyper | Body |
| **chr19_3435748** | cg12180253 | 0,032133026 | hyper | 0,066472641 | hyper | Body |
| **chr19_3443884** | cg10260031 | 0,002465771 | hyper | 0,026818977 | hyper | Body |
| **chr19_3449026** | cg17852172 | 0,001935268 | hyper | 0,02405464 | hyper | Body |
| **chr19_3455164** | cg27558864 | 0,016600122 | hyper | 0,015639092 | hyper | Body |
| **chr19_3456330** | cg21584759 | 0,004136373 | hyper | 0,066321636 | hyper | Body |
| **chr19_3458196** | cg04251208 | 0,023695038 | hyper | 0,020466517 | hyper | Body |
| ***NR4A2*** | | | | | | |
| **chr2_156329379** | cg11358945 | 0,036550108 | hyper | 0,110820999 | hyper | Body |
| **chr2_156329800** | cg00194126 | 0,066950976 | hyper | 0,207423034 | hyper | Body |
| **chr2_156329809** | cg14811105 | 0,037489246 | hyper | 0,107825557 | hyper | Body |
| **chr2_156330717** | cg11399967 | 0,003590016 | hyper | 0,10838241 | hyper | 5'UTR |
| **chr2_156331917** | cg11932911 | 0,011700643 | hyper | 0,001727676 | hyper | 5'UTR |
| **chr2_156332453** | cg09408520 | 0,00113489 | hyper | 0,011725976 | hyper | 5'UTR |
| **chr2_156332796** | cg20804199 | 0,005696761 | hyper | 0,011421612 | hyper | TSS200 |
| **chr2_156332802** | cg18881247 | 0,009801645 | hyper | 0,002161978 | hyper | TSS200 |
| **chr2_156332998** | cg20945253 | 0,001205438 | hyper | 0,014881165 | hyper | TSS1500 |
| ***GRM2*** | | | | | | |
| **chr3_51706185** | cg13139998 | 0,037227186 | hyper | 0,004276304 | hyper | TSS1500 |
| **chr3_51706606** | cg22739025 | 0,046208222 | hyper | 0,055987652 | hyper | TSS1500 |
| **chr3_51706725** | cg25693132 | 0,07403348 | hyper | 0,06703806 | hyper | TSS1500 |
| **chr3_51706834** | cg21899500 | 0,040387903 | hyper | 0,067434078 | hyper | TSS1500 |
| **chr3_51706859** | cg00664406 | 0,055796006 | hyper | 0,067815772 | hyper | TSS1500 |
| **chr3_51706880** | cg04453050 | 0,069668165 | hyper | 0,046509147 | hyper | TSS200 |
| **chr3_51706940** | cg26079664 | 0,124844288 | hyper | 0,121419333 | hyper | TSS200 |
| **chr3_51707119** | cg12934382 | 0,172666469 | hyper | 0,177367136 | hyper | 1stExon;5'UTR |
| **chr3_51707264** | cg00303541 | 0,070776568 | hyper | 0,12863565 | hyper | 5'UTR |
| **chr3_51707457** | cg21213853 | 0,030204657 | hyper | 0,114693903 | hyper | 5'UTR |
| **chr3_51708148** | cg23585673 | 0,015468346 | hyper | -0,049499856 | hypo | 5'UTR |
| **chr3_51708497** | cg26010751 | 0,007735327 | hyper | -0,073901107 | hypo | 5'UTR |
| **chr3_51711305** | cg20984065 | 0,069114981 | hyper | -0,038036151 | hypo | 5'UTR;Body |
| **chr3_51712654** | cg00371841 | 0,008457399 | hyper | -0,013933864 | hypo | 5'UTR;Body |
| **chr3_51712707** | cg25423647 | 0,016862312 | hyper | 0,00025017 | hyper | 5'UTR;Body |
| **chr3_51713082** | cg10707626 | 0,004916665 | hyper | 0,008780438 | hyper | 5'UTR;Body |
| **chr3_51715127** | cg26369050 | 0,00245467 | hyper | -0,014781281 | hypo | 5'UTR;Body |
| **chr3_51715172** | cg11025960 | 0,008914268 | hyper | -0,025527856 | hypo | 5'UTR;Body |
| **chr3_51715781** | cg08063086 | 0,014958737 | hyper | 0,002834741 | hyper | Body |
| **chr3_51715836** | cg02738298 | 0,011860079 | hyper | -0,0423308 | hypo | Body |
| ***PGK1*** | | | | | | |
| **chrX_78103866** | cg00151234 | 0,047678093 | hyper | 0,36441522 | hyper | TSS1500 |
| **chrX_78103953** | cg13203541 | 0,095779254 | hyper | 0,378778484 | hyper | TSS1500 |
| **chrX_78104038** | cg00832270 | 0,07909913 | hyper | 0,349211537 | hyper | TSS200 |
| **chrX_78104047** | cg15418221 | 0,062814207 | hyper | 0,350105434 | hyper | TSS200 |
| **chrX_78104056** | cg07781082 | 0,0361132 | hyper | 0,299258144 | hyper | TSS200 |
| **chrX_78104063** | cg09790289 | 0,169716238 | hyper | 0,222119742 | hyper | TSS200 |
| **chrX_78104067** | cg14794494 | 0,122391298 | hyper | 0,220376045 | hyper | TSS200 |
| **chrX_78104116** | cg26744454 | 0,083704345 | hyper | 0,257236177 | hyper | TSS200 |
| **chrX_78104242** | cg24657313 | 0,080492023 | hyper | 0,343179674 | hyper | 1stExon;5'UTR |
| **chrX_78104363** | cg16181613 | 0,050350515 | hyper | 0,103397543 | hyper | 1stExon |
| **chrX_78104379** | cg12642155 | 0,028879309 | hyper | 0,038256637 | hyper | 1stExon |
| **chrX_78104427** | cg20203466 | 0,096415434 | hyper | 0,177075412 | hyper | Body |
| **chrX_78105013** | cg08955859 | 0,09541345 | hyper | 0,184085753 | hyper | Body |
| **chrX_78105612** | cg25409213 | 0,070260304 | hyper | 0,169068455 | hyper | Body |
| **chrX_78105706** | cg18272390 | 0,126129243 | hyper | 0,079071141 | hyper | Body |
| ***STMN1*** | | | | | | |
| **chr1_25900158** | cg07501506 | -0,006607795 | hypo | -0,025388903 | hypo | 3'UTR;Body |
| **chr1_25905182** | cg03539758 | -0,007878579 | hypo | -0,02121715 | hypo | 5'UTR |
| **chr1_25905820** | cg22943243 | -0,005481681 | hypo | -0,005767929 | hypo | 5'UTR |
| **chr1_25905974** | cg23079732 | -0,0016544 | hypo | -0,004420894 | hypo | 5'UTR;1stExon |
| **chr1_25906235** | cg19400000 | -0,005781503 | hypo | -0,004984153 | hypo | 5'UTR;TSS200;5'UTR |
| **chr1_25906841** | cg03210277 | -0,00862244 | hypo | -0,014338367 | hypo | 1stExon;TSS1500;5'UTR |
| **chr1_25907524** | cg09518245 | -0,001726419 | hypo | -0,018879971 | hypo | TSS1500 |
| ***LIMS2*** | | | | | | |
| **chr2_127643267** | cg24879690 | -0,033479463 | hypo | -0,044462971 | hypo | Body;5'UTR |
| **chr2_127643965** | cg01638636 | -0,025914222 | hypo | -0,00915249 | hypo | Body;5'UTR |
| **chr2_127645568** | cg25279605 | -0,004456273 | hypo | -0,008562933 | hypo | TSS1500;Body;5'UTR |
| **chr2_127645711** | cg20074593 | -0,019579421 | hypo | -0,04175199 | hypo | TSS200;Body |
| **chr2_127645723** | cg12979562 | -0,01163105 | hypo | -0,016156837 | hypo | TSS200;Body;5'UTR |
| **chr2_127645739** | cg27522311 | -0,023568131 | hypo | -0,036306474 | hypo | TSS200;Body;5'UTR |
| **chr2_127645867** | cg27298008 | -0,011244587 | hypo | -0,098226479 | hypo | 1stExon;Body;5'UTR |
| **chr2_127645915** | cg19604027 | -0,024658245 | hypo | -0,050673153 | hypo | 1stExon;Body;5'UTR |
| **chr2_127645973** | cg12662563 | -0,021196221 | hypo | -0,066483379 | hypo | 1stExon;Body;5'UTR |
| **chr2_127652455** | cg05361273 | -0,114666508 | hypo | -0,029596006 | hypo | 3'UTR;Body |
| **chr2_127653115** | cg03022211 | -0,00342936 | hypo | -0,033661744 | hypo | Body |
| **chr2_127653444** | cg15730793 | -0,039293481 | hypo | -0,043379617 | hypo | Body |
| **chr2_127655435** | cg16362481 | -0,051759191 | hypo | -0,0586286 | hypo | Body |
| **chr2_127655931** | cg13728327 | -0,033065874 | hypo | -0,000680267 | hypo | Body |
| **chr2_127656094** | cg19984474 | -0,002257971 | hypo | -0,029371994 | hypo | Body |
| **chr2_127662076** | cg01761729 | -0,036780325 | hypo | -0,004241898 | hypo | Body;5'UTR |
| **chr2_127664636** | cg20329510 | -0,013682953 | hypo | -0,011142756 | hypo | TSS200;Body;5'UTR |
| **chr2_127664650** | cg19426955 | -0,012214136 | hypo | -0,02699787 | hypo | Body;TSS200;5'UTR |
| **chr2_127664710** | cg11535366 | -0,006026014 | hypo | -0,054014783 | hypo | Body;TSS200;5'UTR |
| **chr2_127672142** | cg22490845 | -0,006251313 | hypo | -0,068126434 | hypo | Body;5'UTR |
| **chr2_127673363** | cg04947052 | -0,019162414 | hypo | -0,040141695 | hypo | Body;5'UTR |
| **chr2_127674355** | cg19479517 | -0,024180468 | hypo | -0,007586919 | hypo | Body;5'UTR |
| **chr2_127674436** | cg06192618 | -0,01051799 | hypo | -0,040357574 | hypo | Body;5'UTR |
| **chr2_127674477** | cg05855166 | -0,00124141 | hypo | -0,055736867 | hypo | Body;5'UTR |
| **chr2_127675036** | cg17976576 | -0,00034058 | hypo | -0,008943471 | hypo | 1stExon;5'UTR |
| **chr2_127676995** | cg09369262 | -0,013937305 | hypo | -0,047255386 | hypo | 5'UTR |
| **chr2_127677555** | cg20264452 | -0,001706407 | hypo | -0,011820769 | hypo | 5'UTR |
| **chr2_127679143** | cg16477975 | -0,006901998 | hypo | -0,0536065 | hypo | 5'UTR |
| **chr2_127681771** | cg03689092 | -0,01782782 | hypo | -0,026912229 | hypo | 1stExon;5'UTR |
| **chr2_127682469** | cg18217192 | -0,004165822 | hypo | -0,019666352 | hypo | TSS1500 |
| **chr2_127682976** | cg09177541 | -0,007067056 | hypo | -0,017811014 | hypo | TSS1500 |

*Legend:* Each raw indicates the unique chromosomal coordinates, the diff.meth values for common detected dmCpGs between Array 1 and Array 2, and the genomic localization for the ten network-oriented DMGs.

**Supplementary Table 13**.Characteristics of validation set (qRT-PCR)

| **Variable** | **PAH (N=20)** | **CTRLs (N=10)** |
| --- | --- | --- |
| **Age, y** | 63.8 ± 14.6 | 46.4 ± 11.8 |
| **Female** | 12 (60.0) | 6 (60.0) |
| **PAH phenotype** |  |  |
| **IPAH** | 10 (50.0) |  |
| **PAH-SSc** | 4 (20.0) |  |
| **PAH-CHD** | 4 (20.0) |  |
| **POPH** | 2 (10.0) |  |
| **NYHA class** |  |  |
| **I** | 1 (5.0) |  |
| **II** | 9 (45.0) |  |
| **III** | 10 (50.0) |  |
| **Invasive hemodynamics** |  |  |
| **RAP, mmHg** | 5.10 ± 3.71 |  |
| **mPAP, mmHg** | 36.4 ± 12.5 |  |
| **PCWP, mmHg** | 9.15 ± 2.54 |  |
| **PVR, WU** | 6.44 ± 4.61 |  |
| **CI, L/min/m2** | 2.77± 0.79 |  |
| **Risk category** |  |  |
| **Low-risk** | 9 (45.0) |  |
| **Intermediate-risk** | 4 (20.0) |  |
| **High-risk** | 7 (35.0) |  |

Data are n (%) or mean ± standard deviation (SD). *Abbreviations:* CI: Cardiac Index; CTRL: Healthy Control; IPAH: Idiopathic PAH; mPAP: Mean Pulmonary Arterial Pressure; NYHA: New York Heart Association; PAH-CHD: PAH Associated with Congenital Heart Disease; PAH-SSc: PAH Associated with Systemic Sclerosis: PCWP: Pulmonary Capillary Wedge Pressure; POPH: PAH Associated with Portal Hypertension; PVR: Pulmonary Vascular Resistance; RAP: Right Atrial Pressure; RRBS: Reduced Representation Bisulfite Sequencing

**Supplementary Table 14**.Characteristics of validation set (WB)

| **Variable** | **PAH (N=12)** | **CTRLs (N=5)** |
| --- | --- | --- |
| **Age, y** | 63.8 ± 14.6 | 46.4 ± 13.9 |
| **Female** | 12 (60.0) | 3 (60.0) |
| **PAH phenotype** |  |  |
| **IPAH** | 5 (41.7) |  |
| **PAH-SSc** | 4 (33.3) |  |
| **POPH** | 2(16.7) |  |
| **PAH-CHD** | 1 (8.3) |  |
| **NYHA class** |  |  |
| **I** | 1 (8.3) |  |
| **II** | 6 (50.0) |  |
| **III** | 5 (41.7) |  |
| **Invasive hemodynamics** |  |  |
| **RAP, mmHg** | 7.58 ± 8.16 |  |
| **mPAP, mmHg** | 35.3 ± 9.27 |  |
| **PCWP, mmHg** | 9.75 ± 3.02 |  |
| **PVR, WU** | 4.88 ± 2.65 |  |
| **CI, L/min/m2** | 3.08± 1.0 |  |
| **Risk category** |  |  |
| **Low-risk** | 6 (50.0) |  |
| **Intermediate-risk** | 1 (8.3) |  |
| **High-risk** | 5 (41.7) |  |

Data are n (%) or mean ± standard deviation (SD). *Abbreviations:* CI: Cardiac Index; CTRL: Healthy Control; IPAH: Idiopathic PAH; mPAP: Mean Pulmonary Arterial Pressure; NYHA: New York Heart Association; PAH-CHD: PAH Associated with Congenital Heart Disease; PAH-SSc: PAH Associated with Systemic Sclerosis: PCWP: Pulmonary Capillary Wedge Pressure; POPH: PAH Associated with Portal Hypertension; PVR: Pulmonary Vascular Resistance; RAP: Right Atrial Pressure; RRBS: Reduced Representation Bisulfite Sequencing

**Supplementary Figure 1**. Histogram of % CpG methylation *per* sample


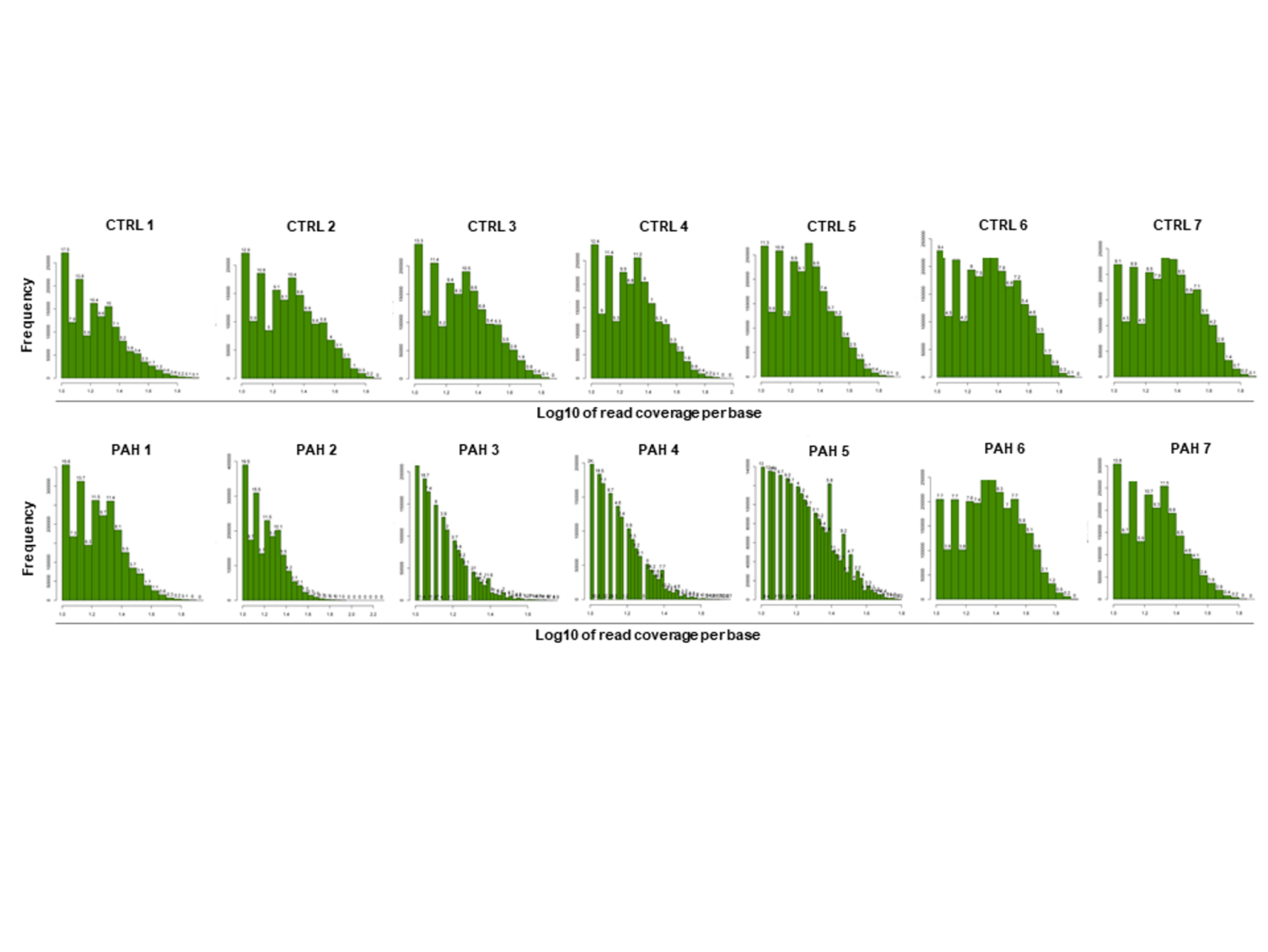


*Abbreviations:* CTRL: Healthy Control; PAH: Pulmonary Arterial Hypertension.

**Supplementary Figure 2**. Histogram of CpG coverage *per* sample


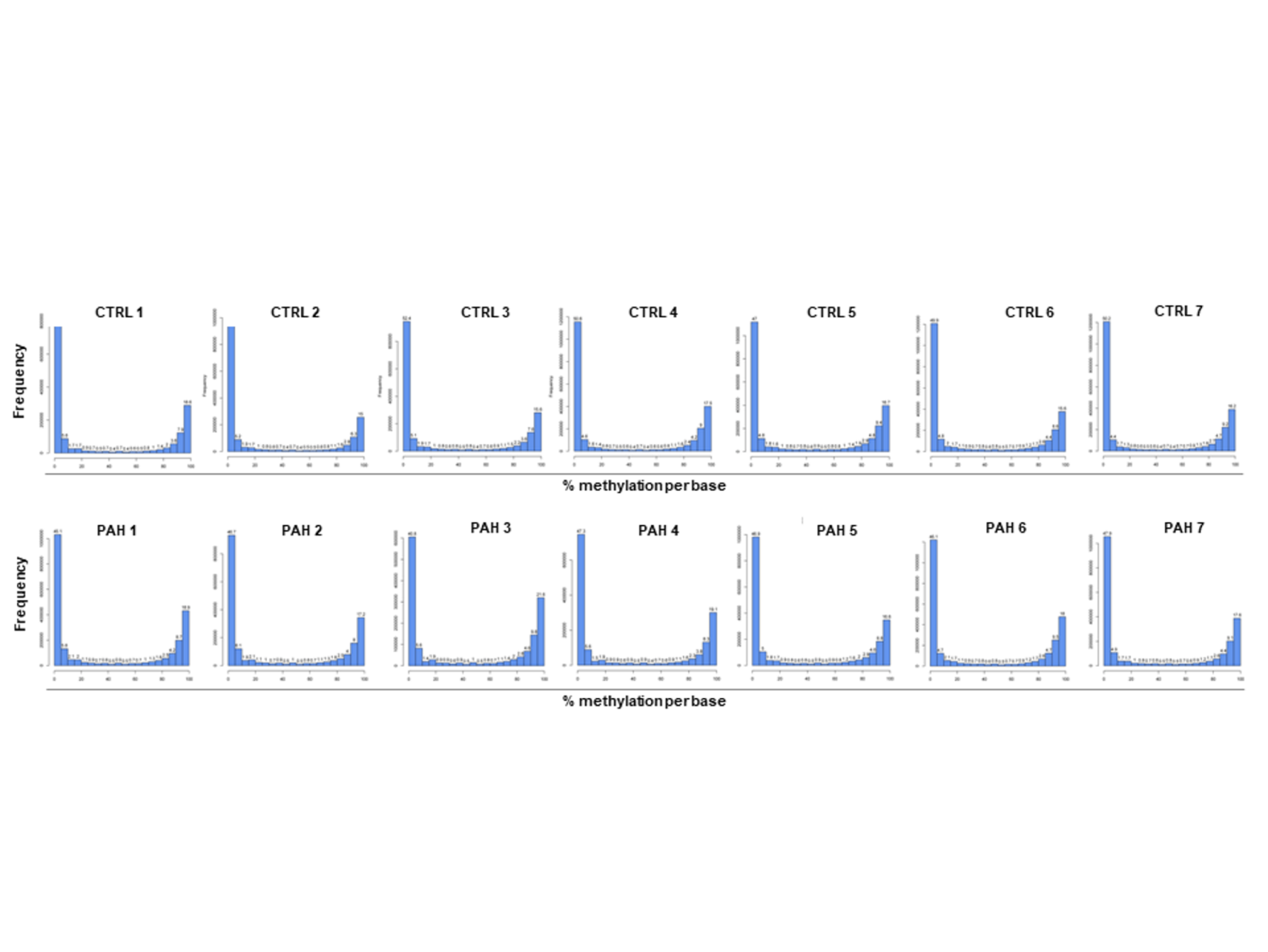


*Abbreviations:* CTRL: Healthy Control; PAH: Pulmonary Arterial Hypertension.

**Supplementary Figure 3**.Power analysis of the differential methylation analysis for annotated genes

**
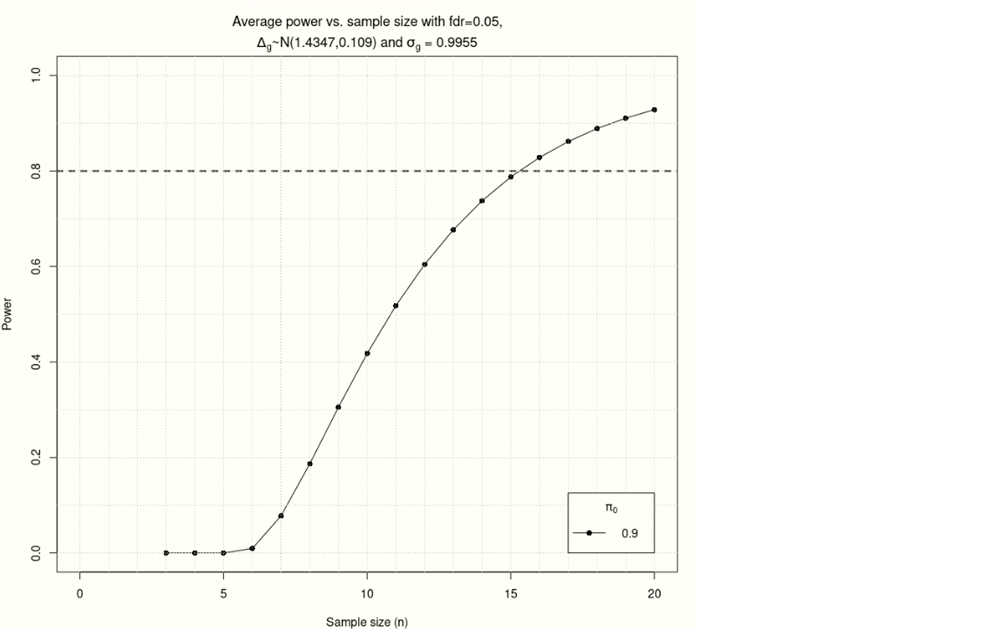
**

**Legend**:The estimated power is based on simulation method using a negative binomial distribution. The result indicates that with a total sample size of 14, which is what we used in the study, we reported an achieved power of 0.74 at FDR= 0.05.

**Supplementary Figure 4**.Genomic localization of dmCpGs


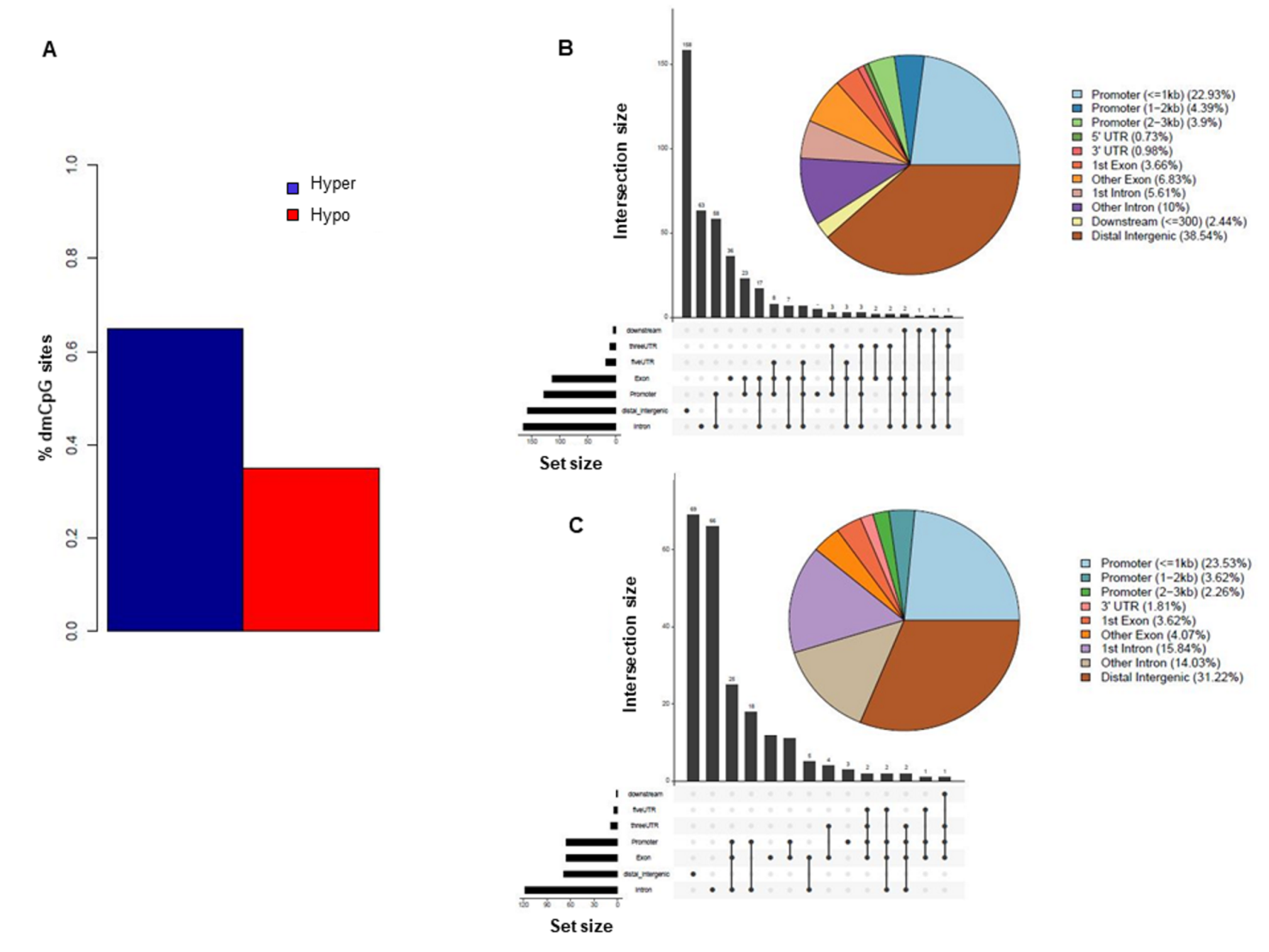


**Legend:** (**A**)The bar plot represents the percentage of hypermethylated *vs.* hypomethylated dmCpGs in circulating CD4^+^ T cells of patients with PAH *vs.* controls.The pie-charts represent the distribution of dmCpGs relative to gene regions for hypermethylated and hypomethylated genes in (**B**) and (**C**), respectively. The upset plots summarize the overlap of dmCpGs between different gene regions. In each panel, the bottom left horizontal barplot (labelled as Set Size) shows the total number of dmCpGs annotated for each gene region. A single dot in the lower panel indicates the number of dmCpGs that are unique to the corresponding gene region whereas joining dots indicate the number of dmCpGs that are shared between the gene regions. The top barplot in each panel summarizes the number of dmCpGs, each in unique or overlapping combination. In each panel, the pie chart shows the percentage of dmCpGs located in gene regions.*Abbreviations:*dmCpGs: Differentially Methylated CpG Sites; PAH: Pulmonary Arterial Hypertension.

**Supplementary Figure 5**.Chromosomal and CGI-related distribution of dmCpGs


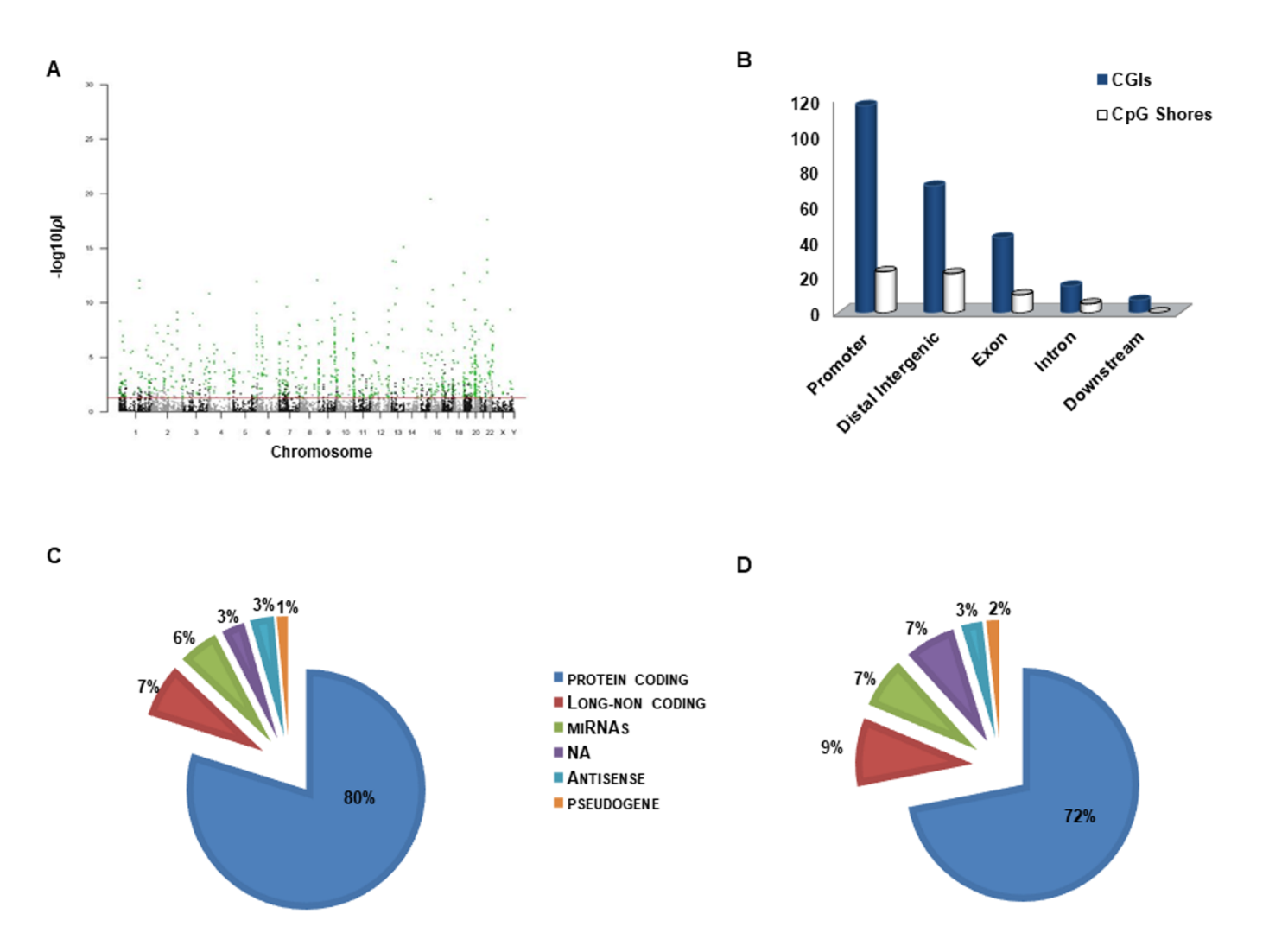


**Legend:** (**A**) Manhattan plot shows the genomic distribution of PAH-associated dmCpGs across all human chromosome regions. The x- axis is the chromosomal position, and the y- axis is the significance on a -log10 scale. Threshold for genome- wide significance (red line) is q-val< 5%. Green points identify dmCpGs defined as regions with more than 25% methylation differences (|ΔM|) and q-val< 0.05. The bar plot (**B**) represents the distribution of dmCpGs in the context of CGIs and CpG shores. The majority of dmCpGs are located in CGIs associated with promoter regions with a prominent percentage of hypermethylated cytosine bases. In (**C**)and(**D**), the pie-charts illustrate that most hypermethylated and hypomethylated DMGs, respectively, encode for proteins.*Abbreviations:* CGIs: CpG Islands; dmCpGs: Differentially Methylated CpG Sites; DMGs: Differentially Methylated Genes; PAH: Pulmonary Arterial Hypertension.

**Supplementary Figure 6**. Hypermethylated lung-specific DNA methylation-PPI subnetwork


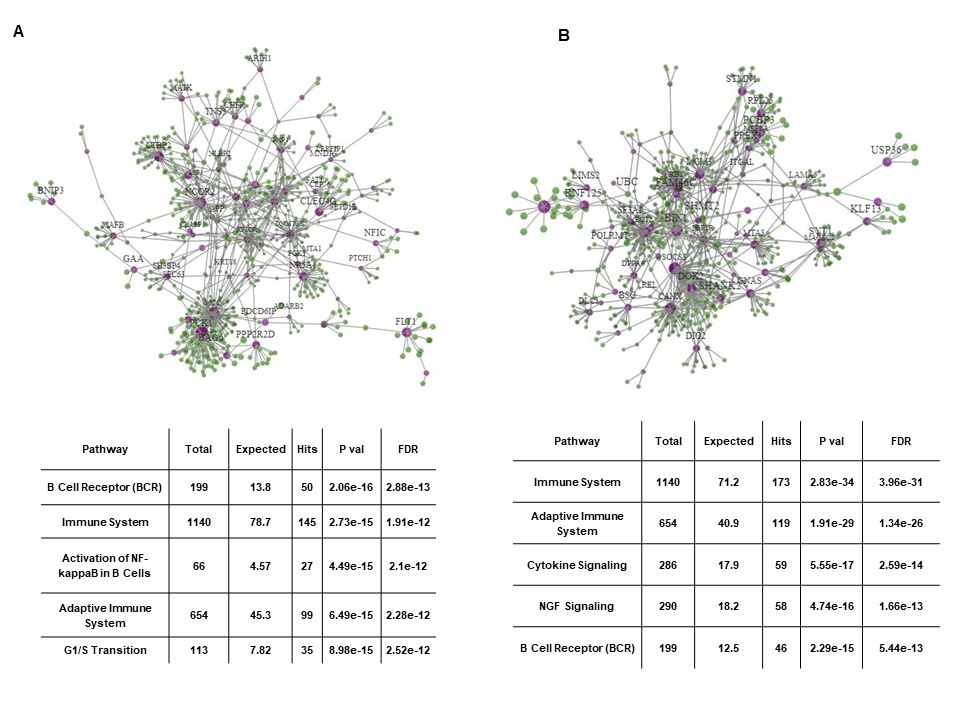


**Legend:** The violet circles represent seeds (hyper-DMGs) and green circles represent interactor nodes, which are linked through PPIs to seeds. The size of each node is scaled according to the number of edges (grey lines). The box below indicates that the top five significant pathways are enriched in immune system functions and cell cycle signaling.*Abbreviations:* DMGs: Differentially Methylated Genes; PAH: Pulmonary Arterial Hypertension; PPIs: Protein-Protein Interactions.

**Supplementary Figure 7**. Hypomethylated lung-specific DNA methylation-PPI subnetwork


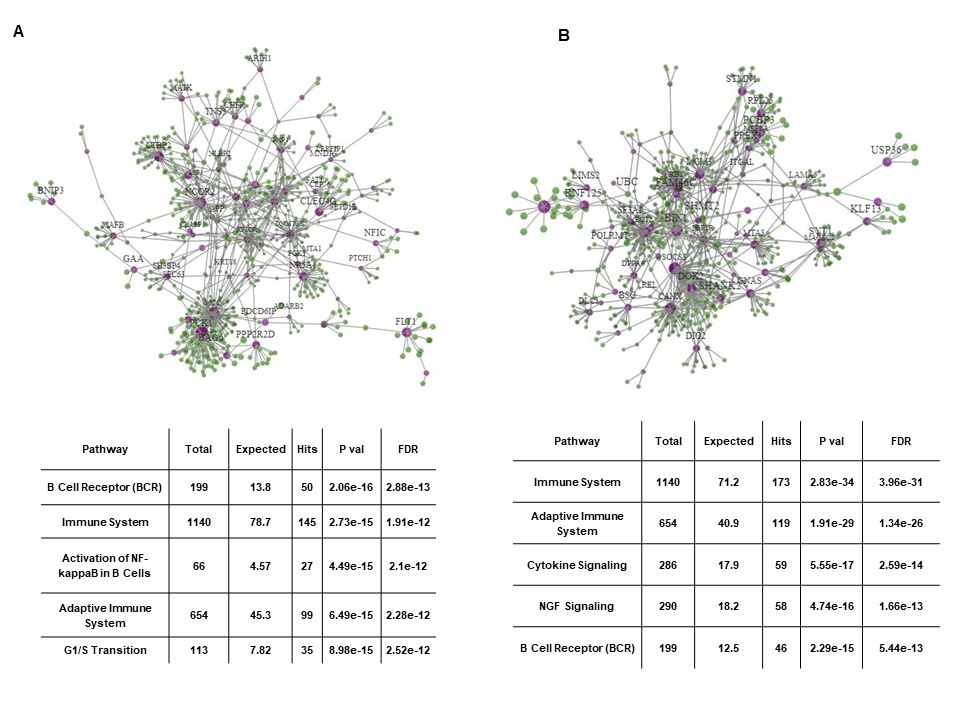


**Legend:** The violet circles represent seeds (hypo-DMGs) and green circles represent interactor nodes, which are linked through PPIs to seeds. The size of each node is scaled according to the number of edges (grey lines). The box below indicates that the top five significant pathways are enriched in immune system functions and growth factor signaling.*Abbreviations:* DMGs: Differentially Methylated Genes; PAH: Pulmonary Arterial Hypertension; PPIs: Protein-Protein Interactions.

**Supplementary Figure 8.** Validation of network-oriented DMGs


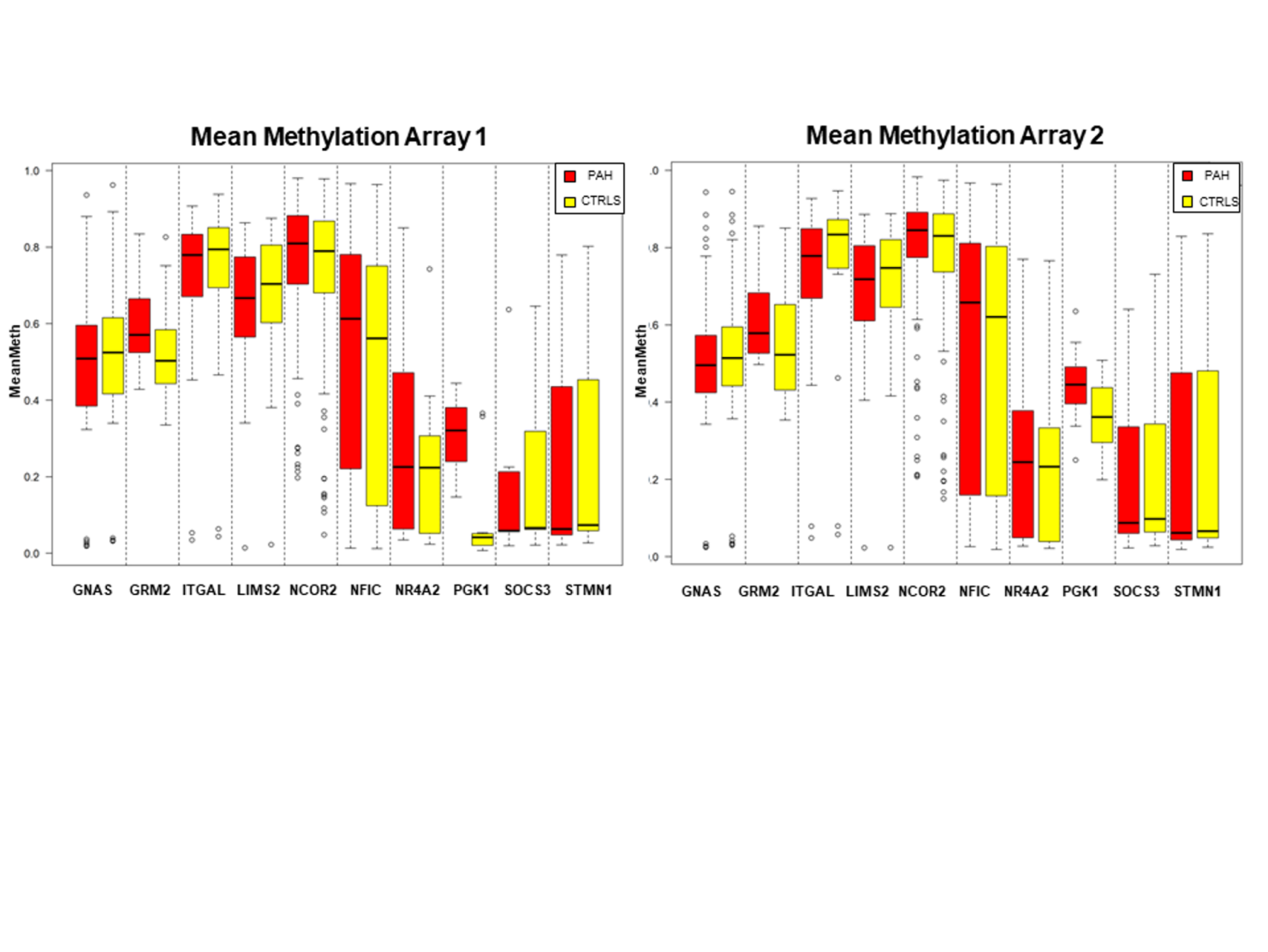


**Legend:** Box plot showing mean methylation pattern for 2 Infinium Human Methylation EPICBeadChip arrays between control (yellow) and PAH (red) biospecimens for the 5 hub DMGs and 5 non hub DMGs. Bottom and top of the box are the 25th and 75th percentile (the lower and upper quartiles, respectively), and the band near the middle of the box is the 50th percentile (the median).*Abbreviations:*CTRLS: Healthy Controls; *ITGAL*: Integrin Subunit Alpha L; *GNAS*: Guanine Nucleotide Binding Protein (G Protein), Alpha Stimulating Activity; *GRM2*: Glutamate Metabotropic Receptor 2; *LIMS2*: LIM Zinc Finger Domain Containing 2; *NCOR2*: Nuclear Receptor Corepressor 2; *NFIC*: Nuclear Factor I C; *NR4A2*: Nuclear Receptor Subfamily 4 Group A Member 2; PAH: Pulmonary Arterial Hypertension; *PGK1*: Phosphoglycerate Kinase 1; *SOCS3*: Suppressor of Cytokine Signaling 3; *STMN1*: Stathmin.

**Supplementary Figure 9.** Original Western Blot image


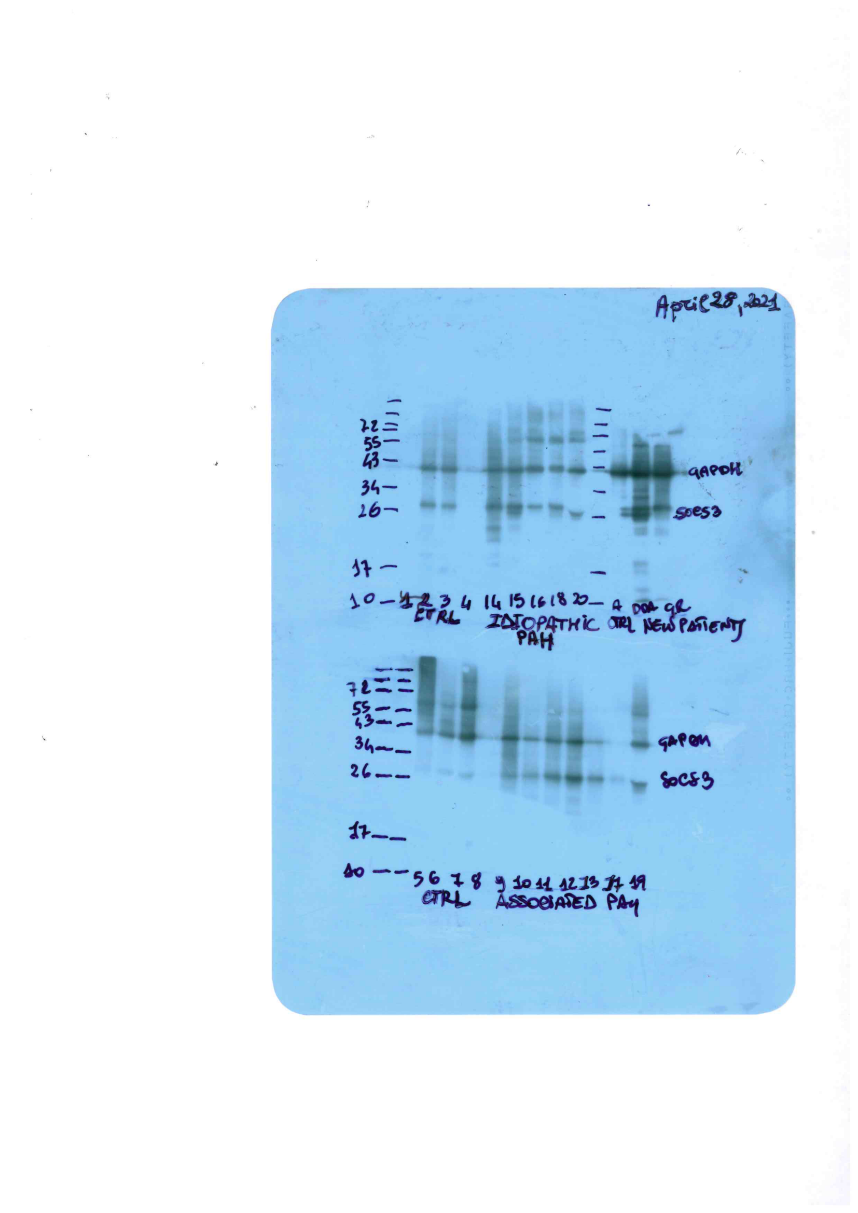


**Supplementary Figure 10.** Linear regression analysis

**
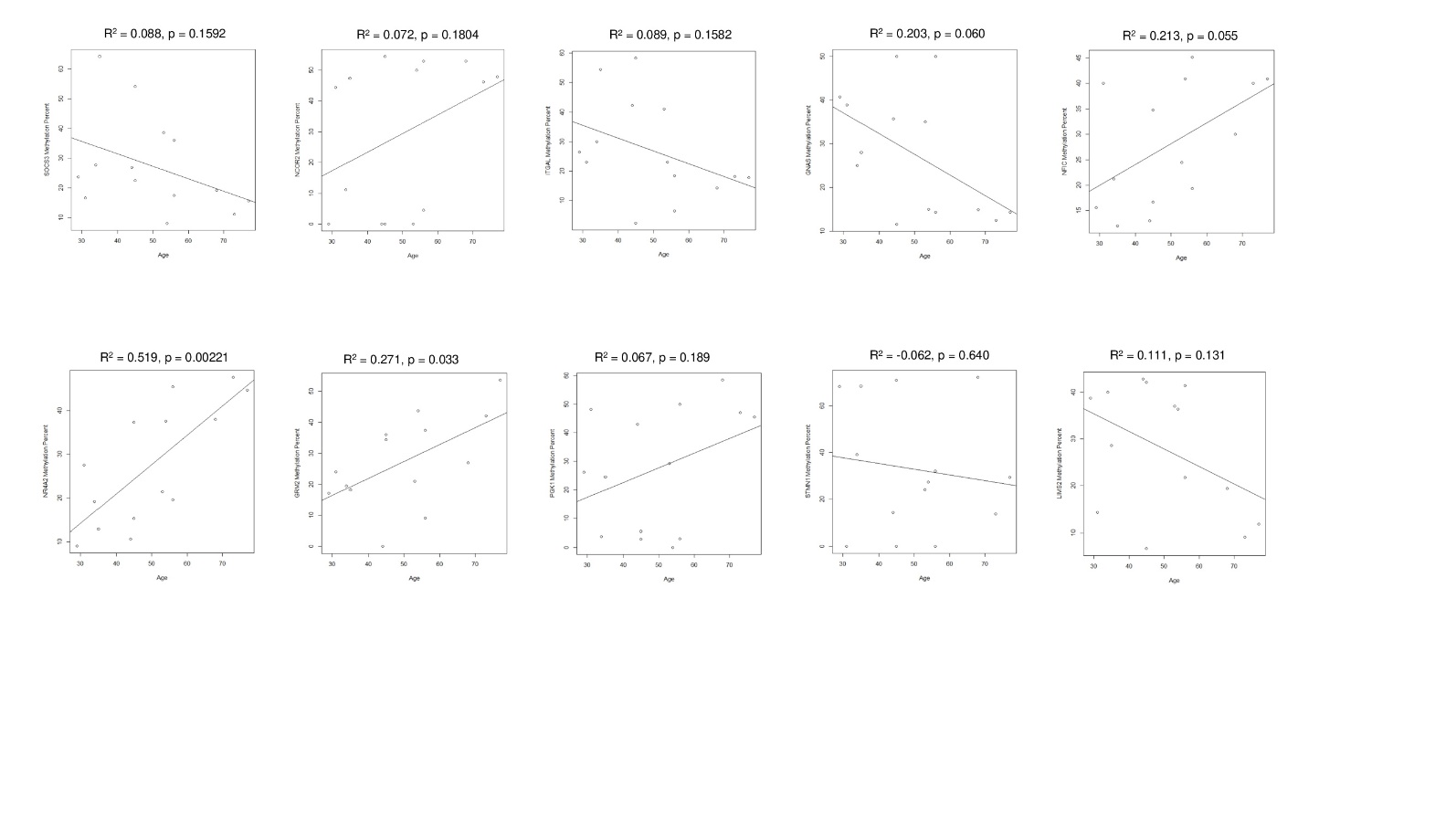
**
